# ICAM2 initiates trans-blood-CSF barrier migration and stemness properties in leptomeningeal metastasis of triple-negative breast cancer

**DOI:** 10.1038/s41388-023-02769-5

**Published:** 2023-07-19

**Authors:** Jhih-Kai Pan, Wen-Der Lin, Yao-Lung Kuo, Yu-Chia Chen, Zhu-Jun Loh, Forn-Chia Lin, Hui-Chuan Cheng, Michael Hsiao, Pei-Jung Lu

**Affiliations:** 1 Institute of Clinical Medicine, College of Medicine, National Cheng Kung University, Tainan, Taiwan; 2 Department of General Surgery, National Cheng Kung University Hospital, Tainan, Taiwan; 3 Division of General Surgery, Department of Surgery, Kaohsiung Veterans General Hospital, Kaohsiung, Taiwan; 4 Department of Surgery, National Cheng Kung University Hospital, College of Medicine, National Cheng Kung University, Tainan, Taiwan; 5 Department of Radiation Oncology, National Cheng Kung University Hospital, Tainan, Taiwan; 6 Genomics Research Center, Academia Sinica, Taipei, Taiwan; 7 Department of Biochemistry, College of Medicine, Kaohsiung Medical University, Kaohsiung, Taiwan; 8 Department of Clinical Medicine Research, National Cheng Kung University Hospital, Tainan, Taiwan

**Keywords:** Breast cancer, Metastasis

## Abstract

Leptomeningeal metastasis (LM) occurs when tumor cells spread to the leptomeningeal space surrounding the brain and the spinal cord, thereby causing poor clinical outcomes. The triple-negative breast cancer (TNBC) has been associated with symptoms of LM and mechanism remained unclear. Through proteomic analysis, we identified high expression of ICAM2 in leptomeningeal metastatic TNBC cells, which promoted the colonization of the spinal cord and resulted in poor survival in vivo. Two-way demonstration indicated that high levels of ICAM2 promoted blood–cerebrospinal fluid barrier (BCB) adhesion, trans-BCB migration, and stemness abilities and determined the specificity of LM in vivo. Furthermore, pull-down and antibody neutralizing assay revealed that ICAM2 determined the specificity of LM through interactions with ICAM1 in the choroid plexus epithelial cells. Therefore, neutralizing ICAM2 can attenuate the progression of LM and prolong survival in vivo. The results suggested that targeting ICAM2 is a potential therapeutic strategy for LM in TNBC.

## Introduction

Leptomeningeal metastasis (LM) occurs when malignant tumor cells infiltrate into the leptomeningeal compartment through hematogenous spread, endoneurial or perineural dissemination along the peripheral nerves, or penetration of the blood–cerebrospinal fluid (CSF) barrier (BCB) [1, 2]. LM is diagnosed among 1–15% of patients with systemic malignancies such as breast cancer (BC), particularly triple-negative breast cancer (TNBC) with characteristics of LM [1, 3, 4]. Currently, patients with LM exhibit poor clinical outcomes, including cognitive disorders and poor likelihood of survival (less than 1 year), and therapeutic strategies are limited [5–8]. Clinical studies have demonstrated that compared with systemic treatment alone, in a randomized phase III trial, intrathecal liposomal cytarabine combined with systemic treatment improved progression-free survival among patients with LM in BC [9, 10]. The safety and efficacy of intrathecal administration of novel agents such as trastuzumab are under evaluation for human epidermal growth factor receptor 2 among patients with LM in BC [11]. However, advanced therapeutic options for patients with LM in TNBC are limited. Therefore, novel therapeutic approaches should be developed to treat LM among patients with TNBC.

The BCB, constituted by tight junction proteins such as claudin-1, is a special structure between blood vessels and nervous tissues in the choroid plexus [12, 13], which produces CSF [14]. The BCB serves as a gatekeeper of the central nervous system (CNS); it prevents the CNS from potentially lethal massive inflammation and toxic substances and maintains ion and glucose homeostasis in the CNS [13, 15]. A previous study demonstrated that tumor cells rapidly populated the choroid plexus and secreted C3a protein, thereby disrupting the structure of the BCB, increasing the permeability of the barrier, and thus resulting in LM [2]. Moreover, tumor cells that rapidly accumulate at the BCB are critical to the progression of LM. In the present study, we identified intercellular adhesion molecule-2 (ICAM2), which mediates the specificity of LM through accumulation on the BCB. ICAM2 is a type I transmembrane glycoprotein containing two immunoglobulin-like domains, which are crucial to lymphocyte recirculation and mediate cell survival signals via pathways associated with LFA-1 [16, 17]. However, the functional role of ICAM2 in tumor progression and metastasis remains unclear.

We identified high levels of ICAM2 that contribute to the specificity of LM in TNBC. We established experimental in vitro and in vivo models of TNBC LM to investigate and characterize the functional role of ICAM2 in TNBC LM. Treatment with anti-ICAM2 neutralizing antibodies can prevent LM in vitro and in vivo and prolong survival in vivo.

## Results

### Establishment of leptomeningeal metastasis TNBC cells and xenograft animal model

**Fig. 1:**
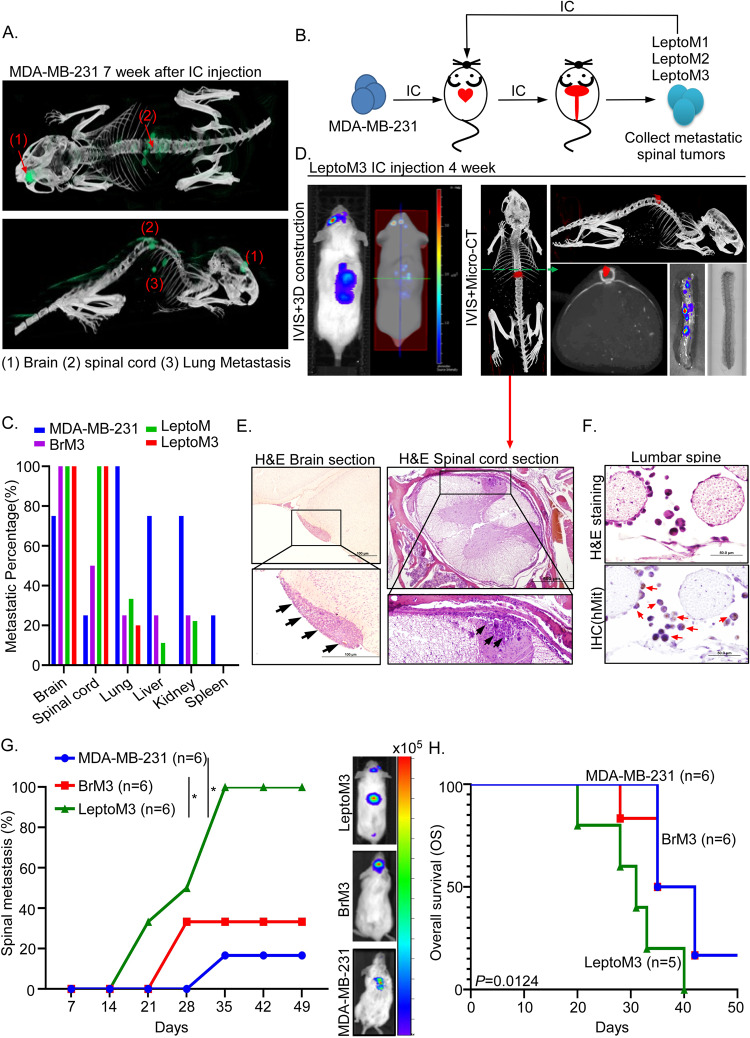
Isolated leptomeningeal-tropic TNBC cells promote the specificity of leptomeningeal metastasis and cause poor survival in vivo. **A** 1 × 10^5^ MDA-MB-231 cells were IC injection in NOD/SCID mice for 7 weeks. Two CNS-metastatic subtypes were detected by IVIS combined with Micro-CT analysis (green signal indicated photon flux signal from IVIS detection). **B** The flowchart of LeptoM (including LeptoM1, LeptoM2, and LeptoM3) cells from in vivo isolation. **C** The metastatic percentage of the different organ metastasis was calculated after 7 weeks IC injection with 1 × 10^5^ MDA-MB-231 (*n* = 4), BrM3 (*n* = 4), LeptoM (*n* = 9), and LeptoM3 (*n* = 5) cells in NOD/SCID mice. **D** Spinal cord colonization of LeptoM3 cells was detected by IVIS combined with Micro-CT analysis (red signal indicated photon flux signal from IVIS detection). **E** The brain and metastatic spinal cord lesions of metastatic LeptoM3 cells were collected and subjected to histopathology analysis (black arrow indicated metastatic lesions) **F** The metastatic LeptoM3 cells in leptomeningeal space were detected by IHC stain. Human mitochondria positive cells (red arrow) indicated injected human tumor cells but not cells origin from mice. **G** The leptomeningeal metastatic ability of MDA-MB-231 cells, BrM3 cells and LeptoM3 cells was evaluated by calculating the percentage of mice with leptomeningeal metastases (*P < 0.05). **H** Kaplan–Meier analysis revealed the overall survival in xenograft mice model. The log-rank (mantel-cox) test was applied to analyze the significance of overall survival between the LeptoM3 group and the MDA-MB-231 group or the LeptoM3 group and BrM3 group. The median survival days of LeptoM3 were 31.2 days, however, the median survival days of BrM3 or MDA-MB-231 were 36.4 days.

Approximately 10–30% of patients with metastatic BC develop CNS metastases. CNS metastasis is clinically classified into two major metastatic phenotypes: parenchymal metastasis [18, 19] and LM. In parenchymal metastasis, tumor cells enter the parenchyma through the endothelium surrounding the vasculature and the connected astrocytes, thereby causing perivascular proliferation [20–22]. In LM, tumor cells enter the CSF compartment and adhere to the pia mater surrounding the brain and spinal cord [2, 8, 23–25]. As revealed by histopathological analysis, our xenograft model demonstrated two major subtypes of CNS-metastatic cells: (1) tumor cells adhered to the blood–brain barrier and (2) tumor cells colonized the pia mater in the entire brain section (Supplementary Fig. S1A). Analysis using the IVIS Spectrum In Vivo Imaging System (IVIS) combined with microcomputed tomography (microCT) revealed MDA-MB-231 cells undergoing obvious colonization in the lung, brain, and spinal cord in the seventh week after IC injection (Fig. 1A); this supported that our xenograft mouse model had two CNS-metastatic subtypes. To investigate the mechanism of leptomeningeal-specific metastasis in TNBC, we established leptomeningeal-tropic TNBC cell lines (LeptoM1, LeptoM2, and LeptoM3) derived from MDA-MB-231 through a series of in vivo selections (Fig. 1B). The photon flux signals represented isolated LeptoM2 and LeptoM3 cells that primarily colonized in the brain and spinal cord but not in the lung or the liver, unlike LeptoM1 cells ex vivo (Supplementary Fig. S1B). The metastatic percentage in multiple organs was calculated by IVIS ex vivo. We detected a 100% specificity of the spinal cord and brain colonization in mice injected with LeptoM3 cells unlike in mice with brain-tropic (BrM3) and MDA-MB-231; however, metastasis was not detected for any other organs (Fig. 1C and Supplementary Fig. S1B). Combined with microCT analysis, IVIS was used to detect the specific location of the photon flux signal in mice. The results indicated that LeptoM3 specifically colonized the spinal cord tissues but did not cause spinal bone erosion (Fig. 1D). Further, the BrM3 cells specifically colonized the brain in mice (Supplementary Fig. S1C). Further, histopathology was used to confirm the LeptoM3 cells infiltrating location in the brain and spinal cord. After 7 weeks of IC injection, the LeptoM3 cells colonized the pia mater in brain sections and adhered to and invaded the nerve tissues in the leptomeningeal space of spinal sections (Fig. 1E). Moreover, immunohistochemical (IHC) staining was used to detect the circulating LeptoM3 cells in the leptomeningeal space of the lumbar spine and those adhered to the nerve tissues (Fig. 1F). Early LM was detected among mice in the third week of injection with LeptoM3 cells, whereas the parental and BrM3 cell signals were undetectable till the fourth or fifth week after injection (Fig. 1G). Compared with the MDA-MB-231 groups, the survival rate of mice injected with LeptoM3 cells was decreased significantly (Fig. 1H; *p* = 0.0124). Likewise, compared with the BrM3 groups, the survival rate of mice injected with LeptoM3 cells was decreased significantly (Fig. 1H; *p* = 0.0242). These results indicated that isolated LeptoM3 cells caused specifically early LM and poor survival in vivo.

### Correlation of high endogenous ICAM2 in TNBC with LM in vitro and in vivo

**Fig. 2:**
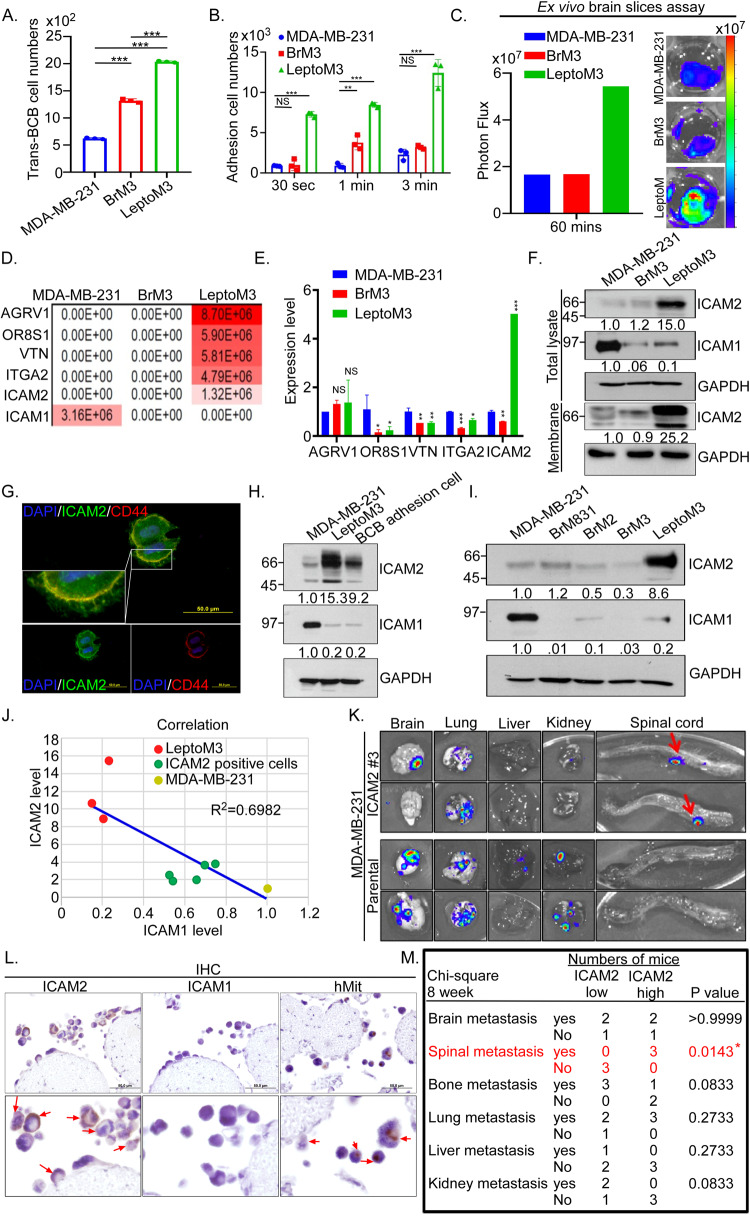
High levels of ICAM2 in TNBC cells are correlated with leptomeningeal metastasis in vitro and in vivo. The leptomeningeal metastasis ability of MDA-MB-231, BrM3 and LeptoM3 cells were investigated using **A** Trans-BCB migration and **B** BCB adhesion assay. 1 × 10^5^ cells were added to plate coated with artificial BCB. After 24 h or 30 sec, 1 min, and 3 min, trans-migrated and adhesive cell numbers were counted by the luciferase activity of cells. **C** The brain adhesion ability of MDA-MB-231, BrM3 and LeptoM3 cells were evaluated by the photon flux represented the cell adhesive ability. **D** The levels of membrane proteins were estimated from the area of the sum of intense tryptic peptides from label-free LC-MS/MS analyses of the different cellular membrane fractions. Enrichment of the membrane proteins are shown. **E** The mRNA levels of *AGRV1, OR8S1, VTN, ITGA2*, and *ICAM2* were validated using qRT-PCR in MDA-MB-231, BrM3, and LeptoM3 cells. **F** Total cell lysates or membrane fractions from MDA-MB-231, BrM3, and LeptoM3 cells were subjected to western blots to investigate the levels of ICAM2 and ICAM1. **G** IF staining of ICAM2 expression (Green) and CD44 (Red) were determined on LeptoM3. Image size was denoted by the scale bar. **H** The protein levels of ICAM2 and ICAM1 were evaluated in BCB adhesive MDA-MB-231 cells compared with MDA-MB-231 and LeptoM3 cells using western blotting. **I** ICAM2 and ICAM1 proteins expression from CNS metastasis TNBC cells were examined by western blots. **J** The correlation of relative protein levels of ICAM1 and ICAM2 in MDA-MB-231, LeptoM3, and ICAM2-positive MDA-MB-231 cells was denoted. **K** 1 × 10^5^ parental or FACS sorting ICAM2-positive #3 MDA-MB-231 cells were IC injection in NOD/SCID mice. After 8 weeks, metastatic lesions in different organs were analyzed using IVIS ex vivo. **L** The expression of ICAM2, ICAM1, and human mitochondria in metastatic tumor cells in the lumbar spine was detected by IHC staining (red arrow indicated signal positive cells). **M** The profiles of different organs metastasis were analyzed by chi-square analysis in high ICAM2 expressive or low ICAM2 expressive TNBC cells-injected mice. (*P < 0.05; **P < 0.01; ***P < 0.001; NS no significant difference).

LM primarily occurs through two main infiltration routes, namely the rapid accumulation of tumor cells in the choroid plexus after hematogenous dissemination [2] and the growth of cancer cells in arachnoid mater and adhered to the pia mater [26]. To investigate the accumulation of tumor cells in the choroid plexus, we constructed an artificial BCB in vitro and tested permeability using fluorescence dye to exclude the cell migration through barrier disruption (Supplementary Fig. S2A, B). The luciferase activities of tumor cells were assessed, and cell numbers were determined in vitro (Supplementary Fig. S2C). The cell numbers of migrated trans-BCB cells increased 4-fold or 1.3-fold in LeptoM3 cells relative to MDA-MB-231 or BrM3, respectively (Fig. 2A). Furthermore, the number of invasive cells significantly increased 8.5-fold in LeptoM3 cells compared with in MDA-MB-231 cells under low serum attracted conditions, mimicking the physiological conditions of CSF (Supplementary Fig. S3A). The isolated LeptoM3 cells notably caused a time-dependent increase in BCB adhesion abilities compared with MDA-MB-231 and BrM3 cells (Fig. 2B). In addition, tumor cells adhered to the pia mater were evaluated using a brain slice adhesion assay [27]. The number of adhesive tumor cells increased 3.4-fold or 3.3-fold in the LeptoM3 cells compared with MDA-MB-231 or BrM3 cells, respectively. The LeptoM3 cells primarily colonized the pia mater in the brain; however, MDA-MB-231 and BrM3 cells primarily colonized the parenchyma of the brain (Fig. 2C and Supplementary Fig. S3B). In LM progression, the isolated LeptoM3 cells enhanced BCB adhesion and trans-BCB migration abilities in vitro. Moreover, we proposed that specific plasma membrane proteins expressed in LeptoM3 cells may cause LM as a result of tumor cell colonization in the choroid plexus.

To identify the specific plasma membrane proteins in LM, proteomic analysis was performed for parental (MDA-MB-231), brain-tropic (BrM3), and leptomeningeal-tropic (LeptoM3) TNBC cells. We then compared the potential plasma membrane proteins and ranked the protein profiles on the basis of two filtering criteria: (1) the proteins should be plasma membrane proteins and (2) the proteins should be detectable only in LeptoM3 cells but not in BrM3 or in MDA-MB-231 cells. As illustrated in Fig. 2D, AGRV1, OR8S1, VTN, ITGA2, and ICAM2 were specifically expressed in the LeptoM3 cells. Moreover, another regulating cell adhesion ICAM1 that belongs to members of the immunoglobulin and integrin supergene families notably decreased in CNS-metastatic cells. The results of qRT-PCR indicated that only *ICAM2* increased notably in LeptoM3 cells. The changes in mRNA levels of *AGRV1*, *OR8S1*, *VTN*, and *ITGA2* were not notable in LeptoM3 cells, and the corresponding molecules were excluded from the potential molecules (listed in Fig. 2E). The results of western blotting revealed that ICAM2 notably increased by 15.0-fold or 25.2-fold in total lysates or the membrane fraction of LeptoM3 compared with in BrM3 and MDA-MB-231 cells, respectively. By contrast, ICAM1 notably decreased in LeptoM3 compared with in BrM3 and MDA-MB-231 cells (Fig. 2F and Supplementary Fig. S4A). Furthermore, in-gel digestion proteomic analysis revealed that ICAM2 is present in the unique band of LeptoM3 cells compared with MDA-MB-231 and BrM3 by silver staining (Supplementary Fig. S4B). The data from IF also indicated that ICAM2 was highly expressed on the surface of LeptoM3 cells but was not detected on the MDA-MB-231 and BrM3 cells (Fig. 2G and Supplementary Fig. S4C). However, ICAM1 was detected on the cell surface of MDA-MB-231 cells (Supplementary Fig. S4C). To investigate the expression levels of ICAM2 in the LeptoM1, LeptoM2, LeptoM3, and MDA-MB-231, Western blotting was conducted. The western blotting data showed that the protein levels of ICAM2 increased 8.4, 10.3, and 22.6 folds in LeptoM1, LeptoM2, and LeptoM3 cells, respectively, compared with MDA-MB-231 cells (Supplementary Fig. S4D). To investigate whether tumor cells accumulated on the choroid plexus, we determined the expression of the ICAM2 and ICAM1 proteins. The BCB-adhered MDA-MB-231 cells were isolated using an in vitro artificial BCB assay (Supplementary Fig. S4E). The BCB-adhered MDA-MB-231 cells exhibited higher levels of ICAM2 and lower levels of ICAM1 than parental MDA-MB-231 cells; this pattern was similar to that observed for in vivo isolated LeptoM3 cells (Fig. 2H). To investigate whether high levels of ICAM2 and low levels of ICAM1 have organotropic metastasis properties, different CNS metastasis tropic cells including BrM-831, BrM3, and LeptoM3 cells were subjected to Western blotting analysis. High levels of ICAM2 and low levels of ICAM1 were observed in leptomeningeal-tropic TNBC cells but not in brain tropic and parental cells (Fig. 2I). To investigate whether high levels of ICAM2 could promote LM, independent clones of ICAM2-positive MDA-MB-231 cells were sorted through flow cytometry [28]. The protein levels of ICAM2 were increased by 1.8–3.7-fold in ICAM2-positive MDA-MB-231 cells compared with parental cells (Supplementary Fig. S4F). Moreover, the protein levels of ICAM1 decreased to 30–50% in ICAM2-positive MDA-MB-231 cells compared with parental cells. The IF results also indicated that ICAM2 was highly expressed on the cell surface of ICAM2-positive MDA-MB-231 cells (Supplementary Fig. S4G–H). Furthermore, the relative levels of ICAM2 were inversely correlated with the levels of ICAM1 in parental, LeptoM3, and ICAM2-sorting TNBC cells (*R*^2^ = 0.6982; Fig. 2J). The ICAM2-positive MDA-MB-231 cells not only notably increased the BCB adhesion ability in vitro (Supplementary Fig. S4I) but also promoted the spinal cord colonization in vivo (Fig. 2K). IHC revealed that tumor cells infiltrating into the leptomeningeal space exhibited high levels of ICAM2, but ICAM1 was not detected (Fig. 2L). Chi-square analysis showed that high levels of ICAM2 MDA-MB-231 cells significantly increased spinal metastasis compared with low levels of ICAM2 MDA-MB-231 cells (*p* = 0.0143) but did not affect other organs in vivo (Fig. 2M). These results demonstrated that high levels of ICAM2 in TNBC cells was correlated with the incidence of LM in vitro and in vivo.

### Two-way demonstration of ICAM2 promoting the LM in vitro and in vivo

**Fig. 3:**
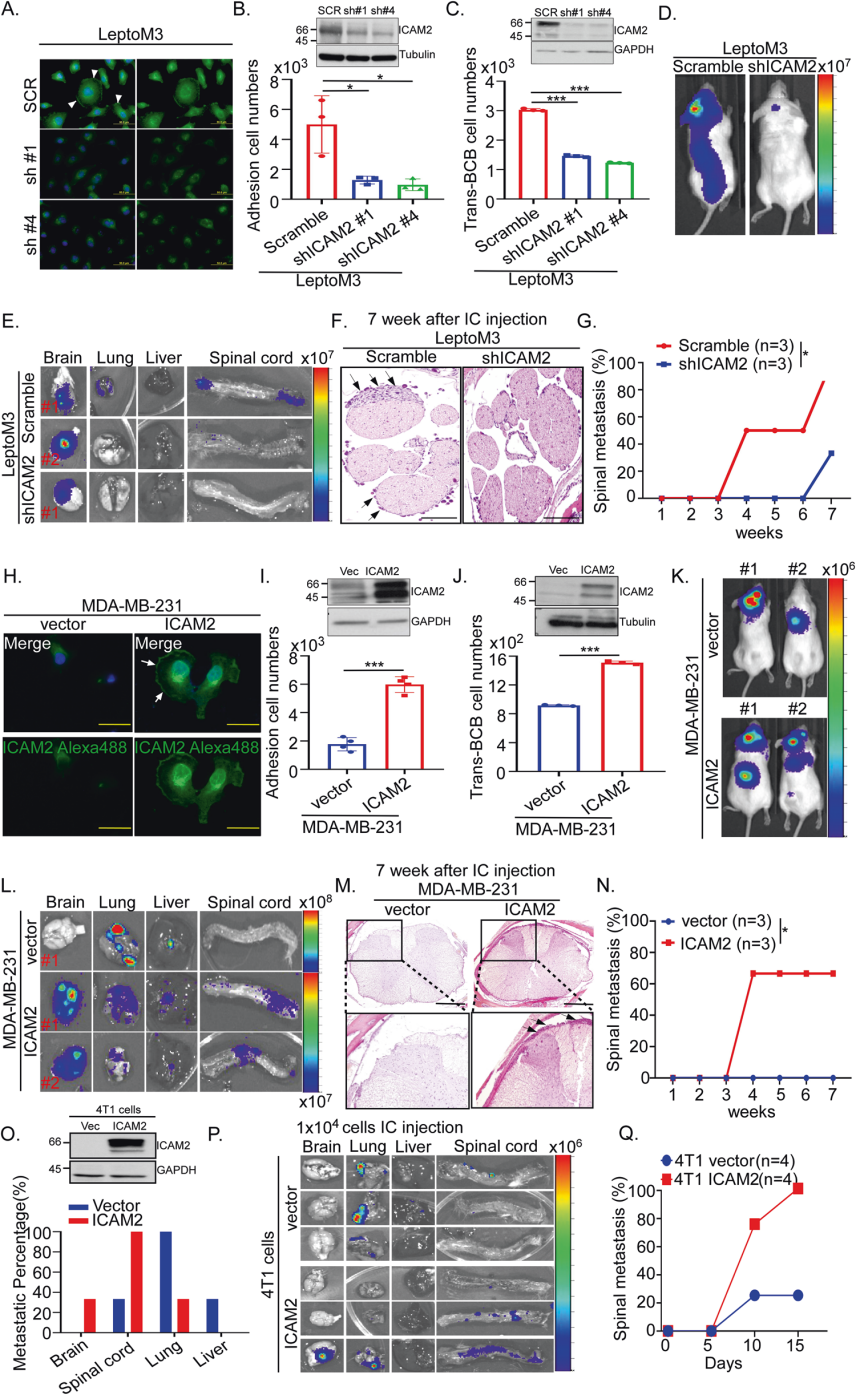
Two-way demonstration of ICAM2 promotes the leptomeningeal metastasis in vitro and in vivo. **A** IF staining of ICAM2 expression (green) was determined on scramble control (SCR) and two independent ICAM2 shRNA knock-down (sh#1 and sh#4) LeptoM3 cells (White arrow indicated that the membrane location of ICAM2). **B** The protein levels of ICAM2 and BCB adhesion ability were investigated in ICAM2 shRNA knock-down LeptoM3 cells compared with scramble control. After 10 min, Adhesive cell numbers were counted by the luciferase activity of cells. **C** The protein levels of ICAM2 and Trans-BCB ability were examined in ICAM2 shRNA knock-down LeptoM3 cells compared with scramble control. After 24 h, transferred cell numbers were counted by the luciferase activity of cells. After 7 weeks of IC injection, **D** Quantitative analysis of scramble control and ICAM2 shRNA knock-down LeptoM3 cells tracing by IVIS **E** Metastatic lesions in different organs were quantitatively analyzed using IVIS ex vivo. **F** The metastatic tumor cells in lumbar spine were detected by H&E staining (Black arrow indicated metastatic tumor cells). **G** The leptomeningeal metastatic ability of scramble control and ICAM2 shRNA knock-down LeptoM3 cells were evaluated by calculating the percentage of mice with leptomeningeal metastases. **H** IF staining of ICAM2 expression (green) was determined on vector control (vector) and ICAM2-overexpressing (ICAM2) MDA-MB-231 cells (White arrow indicated that the membrane location of ICAM2). **I** The protein levels of ICAM2 and BCB adhesion ability were investigated in ICAM2-overexpressing MDA-MB-231 cells compared with vector control. **J** The protein levels of ICAM2 and Trans-BCB migration ability were investigated in ICAM2-overexpressing MDA-MB-231 cells compared with vector control. After 7 weeks of IC injection, **K** Quantitative analysis of vector control and ICAM2-overexpressing MDA-MB231 cells tracing by IVIS. **L** Metastatic lesions in different organs were quantitatively analyzed using IVIS ex vivo. **M** The metastatic tumor cells in leptomeningeal space of spinal cord was determined by H&E staining (Black arrow indicated metastatic tumor cells). **N** The leptomeningeal metastatic ability of vector control and ICAM2-overexpressing MDA-MB-231 cells was evaluated by calculating the percentage of mice with leptomeningeal metastases. **O** The metastatic percentage of the different organ metastasis was calculated after 15 days of IC injection with 1 × 10^4^ ICAM2 overexpressing or vector control 4T1 cells in *BALB*/*c* mice. **P** Metastatic lesions in different organs were quantitatively analyzed using IVIS ex vivo. **Q** The leptomeningeal metastatic ability of vector control and ICAM2-overexpressing 4T1 cells was evaluated by calculating the percentage of mice with leptomeningeal metastases. (*P < 0.05; ***P < 0.001).

To characterize the functional roles of ICAM2 in LM among patients with TNBC, we analyzed ICAM2-overexpressing MDA-MB-231 cells and two independent ICAM2-downregulating LeptoM3 cells. The IF images showed that the plasma membrane location of ICAM2 can be detected in LeptoM3 cells but not in the ICAM2-downregulating LeptoM3 cells (Fig. 3A). The BCB adhesion ability significantly decreased in the ICAM2-downregulating LeptoM3 cells compared with scramble cells (Fig. 3B, *p* < 0.05). The trans-BCB ability significantly decreased in ICAM2-downregulating LeptoM3 cells compared with scramble cells (Fig. 3C, *p* < 0.001). The metastatic lesions of the spinal cord were detected in the scramble group but not in the ICAM2-downregulating group in vivo and ex vivo (Fig. 3D–E). As confirmed by histopathological data, tumor cells suspended in the CSF compartment of the lumbar spine were detected in the scramble group but not in the ICAM2-downregulating group (Fig. 3F). Moreover, LM was first detected at 4 weeks in LeptoM3 cells-injected mice; however, it was undetected until the seventh week in mice in the ICAM2-downregulating group (Fig. 3G and Supplementary Fig. S5A). Furthermore, the IF results showed that the membrane location of ICAM2 was detected in ICAM2-overexpressing MDA-MB-231 cells but not detected in the vector control (Fig. 3H). The results indicated that BCB adhesion ability significantly increased by 3.2-fold (*p* < 0.001) in ICAM2-overexpressing MDA-MB-231 cells compared with the vector control (Fig. 3I). The trans-BCB ability significantly increased by 1.5-fold (*p* < 0.001) in ICAM2-overexpressing MDA-MB-231 cells compared with vector control (Fig. 3J). Most importantly, after 7 weeks of IC injection, the metastatic lesions of the spinal cord were detected in the ICAM2-overexpressing group but not in the vector control group (Fig. 3K–L). Histopathological data demonstrated that tumor cells can infiltrate into the leptomeningeal space that were detected in the ICAM2-overexpressing group but not in the vector control group (Fig. 3M). After 4 weeks of IC injection, LM was detected in 66.6% mice injected with ICAM2-overexpressing MDA-MB-231, whereas it was not detected in the control group until the eleventh week (Fig. 3N and Supplementary Fig. S5B). To assess whether ICAM2 promotes LM in normal background mice model, we constructed the ICAM2-overexpressing 4T1 cells, a breast cancer cell line derived from the mammary gland tissue of a mouse BALB/c strain, for further investigation. Western blotting data showed that the protein levels of ICAM2 significantly were increased in ICAM2-overexpressing 4T1 cells than vector control cells (Fig. 3O). Following, 1 × 10^4^ cells were injected into the left ventricle of BALB/c mice by IC injection following metastasis was monitored by IVIS. After 15 days of IC injection, the percentage of LM was 100% in ICAM2-overexpressing 4T1 bearing mice and 25% in vector control 4T1 bearing mice. Further, 75% of mice with LM were detected on the tenth day in the ICAM2-overexpressing group; however, only 25% of mice with LM were detected in the vector control group (Fig. 3O–Q and Supplementary Fig. S5C). 1 × 10^5^ cells-injected group showed the similar results. After 12 days of IC injection, the percentage of LM was 100% in ICAM2-overexpressing 4T1 bearing mice and 50% in vector control 4T1 bearing mice. Further, 75% of mice with LM were detected on the fifth day in the ICAM2-overexpressing group; however, only 25% of mice with LM were detected at 5 days in the vector control group (Supplementary Fig. S5D–F). These results indicated that ICAM2 promoted early LM in vivo and increased the BCB adhesion and trans-BCB migration in vitro.

### Enhancement of cancer stem cell properties due to high level ICAM2

**Fig. 4:**
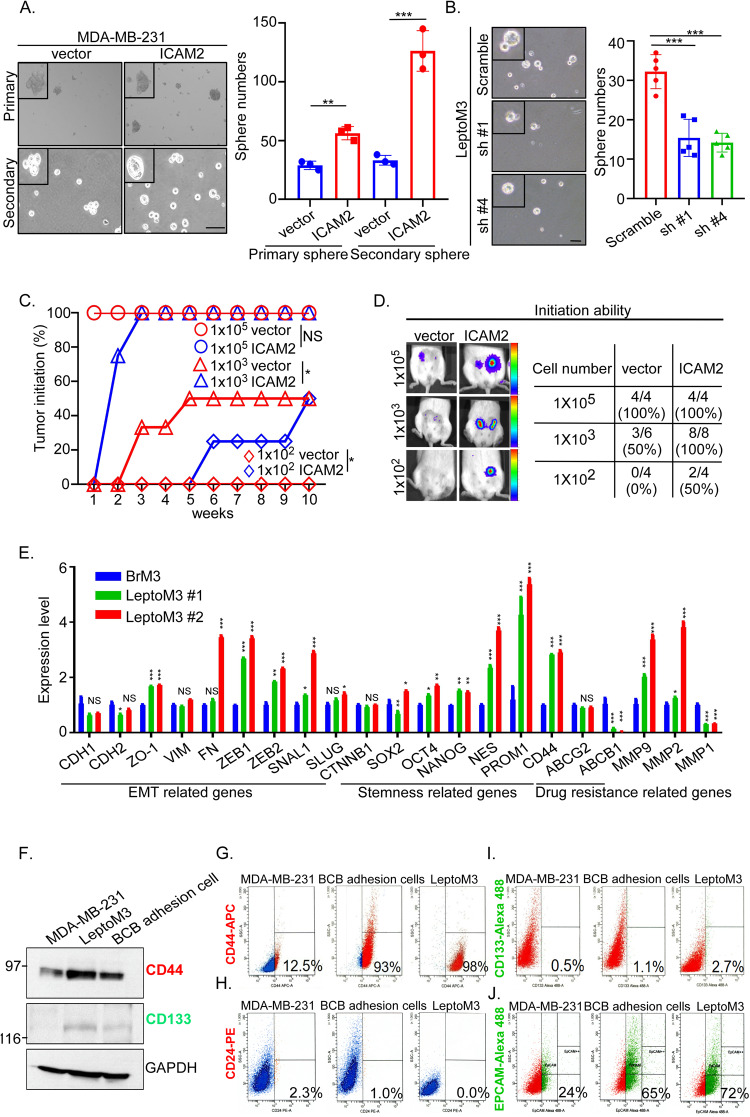
High ICAM2 enhances stemness properties of TNBC cells in vitro and in vivo. **A** ICAM2-overexpressing MDA-MB-231 cells were subjected for primary and secondary sphere forming assay compared with vector control. **B** ICAM2 shRNA knock-down LeptoM3 cells were subjected for primary sphere forming assay compared with scramble control cells. The number of large spheres generated from 1000 tumor cells was calculated on day 10 of culture. **C** The percentage of tumor formation (photon flux signal >1.7 × 10^4^) was calculated in NOD/SCID mice orthotopic injected with vector control or ICAM2-overexpressing MDA-MB-231 cells. **D** Tumor-initiation ability was investigated by different cell numbers orthotopic injection. Quantitative analysis of 1 × 10^5^, 1 × 10^3^, and 1 × 10^2^ vector control (vector) and ICAM2-overexpressing (ICAM2) MDA-MB-231 cells tracing by IVIS at 10 weeks after mammary fat pad injection in NOD/SCID mice. **E** The mRNAs expression of epithelial–mesenchymal transition (EMT)-related genes, stemness genes, and drug resistance genes were examined in two independent LeptoM3 cells compared with BrM3 cells by using qRT-PCR (Three independent replicates of the qRT-PCR expression profile was performed). **F** Expression of stemness markers, CD44 and CD133, were assessed by western blot in MDA-MB-231 cells, LeptoM3, and BCB adhesion cells. Representative immunoblots are shown. The subpopulation of **G** CD44 positive cells, **H** CD24 positive cells **I** CD133 positive cells, and **J** EPCAM-positive cells, were investigated by flow cytometry in MDA-MB-231, LeptoM3, and BCB adhesion cells. Cells were stained with APC-conjugated anti-CD44, PE-conjugated anti-CD24, Alexa488-conjugated anti-CD133, and Alexa488-conjugated anti-EPCAM antibodies. Gating regions were placed based on isotype controls (Three independent replicates of the flow assays were performed). (*P < 0.05; **P < 0.01; ***P < 0.001; NS no significant difference).

In tumor metastasis progression, the stemness and tumor-initiation abilities are crucial for tumor cells to survive environments with poor protein, glucose, and cytokine content and to form secondary tumors [29]. To investigate whether the thousands of high ICAM2 of BCB adhesive and migrated cells from circulation can promote secondary tumor forming in the CSF compartment, two-way demonstrations were performed to determine whether ICAM2 levels could regulate stemness and tumor-initiating abilities. First, the secondary sphere assays were conducted to more accurately measure self-renewal ability in ICAM2 overexpressing and vector control MDA-MB-231 cells. The results indicated that the primary sphere number significantly increased from 28 ± 1.3 to 53 ± 3.4 in ICAM2-overexpressing MDA-MB-231 compared with vector control. Further, we used Accutane dissociating the primary sphere following subjected to secondary sphere assays. The results indicated that the secondary sphere numbers significantly increased from 33 ± 5.2 to 126 ± 19.2 in ICAM2-overexpressing MDA-MB-231 compared with vector control (Fig. 4A). Second, the protein levels of CD44, CD133, CD133^high^, and CD44^high^ TNBC are indicators of cancer stem cell characteristics that promote metastasis [30–32]; the protein levels increased in the ICAM2-overexpressing cells compared with vector control (Supplementary Fig. S6A). Third, the mRNA levels of stemness-related genes including *SOX2*, *NANOG*, *NOTCH1*, and *PROM1* significantly increased in the ICAM2-overexpressing cells compared with vector control (Supplementary Fig. S6B). Fourth, the sphere numbers of ICAM2-downregulated LeptoM3 cells significantly decreased from 32 ± 3.3 to 14 ± 4.8 and 13 ± 1.5 (Fig. 4B, *p* < 0.001). Fifth, the protein levels of CD44 and CD133 decreased in the two independent ICAM2-downregulated LeptoM3 cells compared with scramble cells (Supplementary Fig. S6C). A tumor-initiation ability of 1 × 10^3^ ICAM2-overexpressing groups was detected in the second week after injection, whereas the vector control groups were undetectable till the third week after injection. Six weeks after mammary fat pad injection in the 100 tumor cells, 25% of mice in the ICAM2-overexpressing group reported tumor formation, whereas no tumor was detected in the vector control group until the tenth week (Fig. 4C and Supplementary Fig. S6D). Finally, tumor initiation was evaluated in mice injected with ICAM2-overexpressing cells and vector control cells with mammary fat pad injection; the following tumor-initiation ability was detected using IVIS every week. After 10 weeks of mammary fat pad injection, 1 × 10^3^ and 1 × 10^2^ ICAM2-overexpressing groups exhibited 100% and 50% of tumor-initiation ability, respectively, compared with control groups, which already had 50% and 0% tumor-initiation ability (Fig. 4D). The qRT-PCR results indicated that the mRNA levels of stemness-related genes including *OCT4*, *NANOG*, *NES*, *PROM1 (CD133)*, and *CD44* significantly increased in LeptoM3 cells compared with those in BrM3 cells, indicating that leptomeningeal-tropic TNBC cells acquired CSCs phenotypes (Fig. 4E). CD44 and CD133 proteins significantly increased in the LeptoM3 and BCB adhesion cells, which had high levels of the ICAM2 protein, but not in BBB adhesion cells, which have low ICAM2 protein expression (Fig. 4F and Supplementary Fig. S6E). Previous studies indicated that breast cancer stem cells with specifics markers include CD44^positive^, CD24^negative^, EpCAM ^positive^, and CD133 ^positive^ [33, 34]. The percentage of those markers were evaluated for MDA-MB-231, BCB adhesion, and LeptoM3 cells using flow cytometry. The results showed that the percentage of CD44 positive subpopulation were increased from 12% in MDA-MB-231 to 93% and 98% in BCB adhesion cells and LeptoM3 cells, respectively (Fig. 4G). However, the percentage of CD24 positive subpopulation were decreased from 2.3% in MDA-MB-231 to 1.0% and 0.0% in BCB adhesion cells and LeptoM3 cells, respectively (Fig. 4H). The percentage of CD133 positive subpopulation were increased from 0.5% in MDA-MB-231 to 1.1% and 2.7% in BCB adhesion cells and LeptoM3 cells, respectively (Fig. 4I). The percentage of EpCAM-positive subpopulation were increased from 24% in MDA-MB-231 to 65% and 72% in BCB adhesion cells and LeptoM3 cells, respectively (Fig. 4J). Together, these results indicated that the LeptoM3 and BCB adhesion cells expressed higher stemness markers CD44, CD133, and EPCAM and lower CD24 and the overexpression of ICAM2 promoted sphere formation and tumor initiation.

### ICAM2 interacts with endogenous ICAM1 of choroid plexus cells

**Fig. 5:**
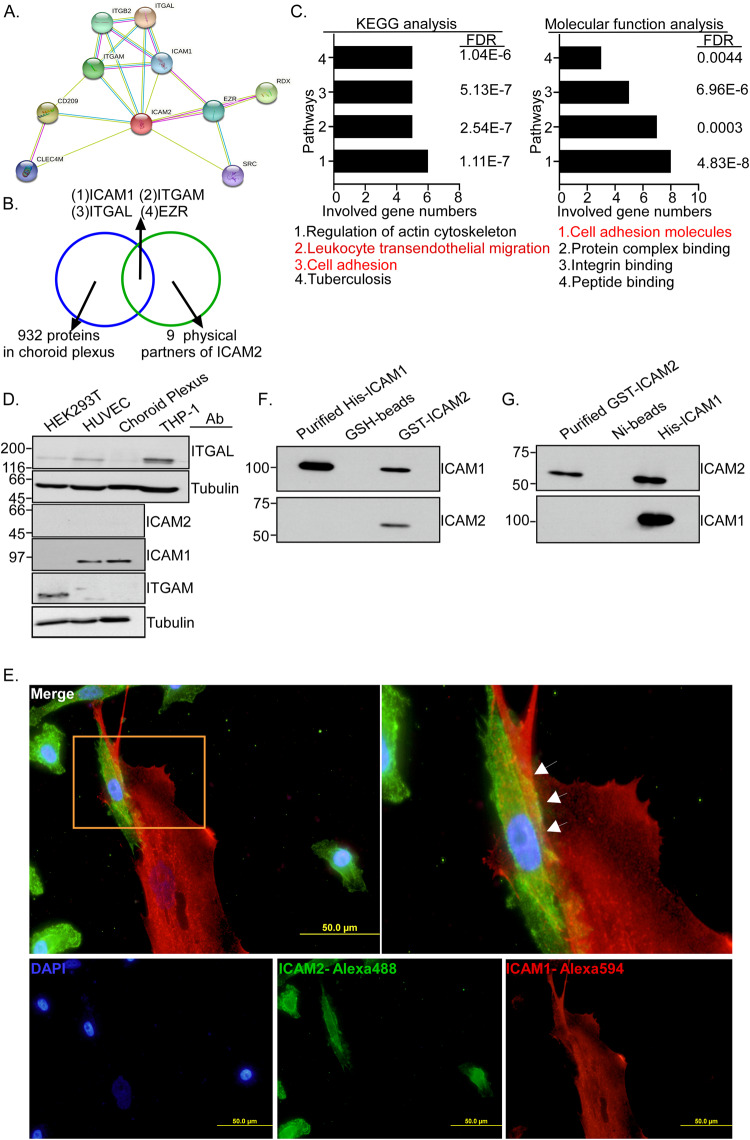
ICAM2 interacts with endogenous ICAM1 of Choroid Plexus cells. **A** Nine physical interaction proteins of ICAM2 were predicted from STRING databases. **B** Four candidates including ICAM1, ITGAM, ITGAL, and EZR were identified through Choroid Plexus Gene Set combined with physical interaction proteins analysis of ICAM2 from STRING databases. **C** Gene ontology functional analysis and KEGG pathway analysis of 9 ICAM2 interacting candidates. **D** The protein levels of ICAM1, ICAM2, ITGAL, and ITGAM were assessed by western blot with ani- ICAM1, ani-ICAM2, anti-ITGAL, and ani-ITGAM antibodies in HEK293T, HUVEC, Choroid Plexus, and THP-1 cells. Representative immunoblots were shown. **E** IF staining of ICAM2 (red) and ICAM1 (green) expression was determined on LeptoM3 and Choroid Plexus cells (White arrow indicated that the interaction interface of LeptoM3 and Choroid Plexus cells). **F** Pull-down assay detected direct binding between purified recombinant ICAM1-His(9x) protein and GST-ICAM2 proteins. The pull-down assay was carried out by incubating GSH-beads with or without GST-ICAM2. After coprecipitation, the purified ICAM1-His(9x) proteins were analyzed by western blotting. **G** Pull-down assay detected direct binding between purified recombinant GST-ICAM2 protein and ICAM1-His(9x) proteins. The pull-down assay was carried out by incubating Ni-beads with or without ICAM1-His(9x). After coprecipitation, the purified GST-ICAM2 proteins were analyzed by western blotting.

To investigate the mechanism of ICAM2-mediated LM in TNBC, we evaluated the physically interacting ICAM2 proteins in the choroid plexus through bioinformatic analysis. Based on the (1) physically interacting partners of ICAM2 predicted from STRING datasets (Fig. 5A, https://string-db.org/) and (2) physically interacting partners of ICAM2 expressed in the choroid plexus derived from the database [35]. Four potential targeting molecules were identified, namely ICAM1, ITGAM, ITGAL, and EZR (Fig. 5B). Through the KEGG pathway and molecular function analysis of nine predicted ICAM2 interacting proteins, the results indicated that cell adhesion and transendothelial migration are crucial pathways for the physical interaction of ICAM2 proteins (Fig. 5C and Supplementary Tables S3 and S4). The Human Protein Atlas datasets (https://www.proteinatlas.org/) show that ICAM1, ITGAM, and ITGAL have tissue specificity. However, the EZR had no tissue specificity expression and was thus excluded from our candidates. Further, western blotting was used to investigate the endogenous protein levels of ICAM1, ITGAM, and ITGAL, which is a subunit of LFA-1, in choroid plexus epithelial cells, HEK293T cells, HUVEC, and THP-1 cells. The ICAM1 exhibited a higher expression in choroid plexus epithelial cells compared with HEK293T and HUVEC cells (Fig. 5D and Supplementary Fig. S7A). The protein levels of ITGAM and ITGAL cannot be detected in choroid plexus epithelial cells; ITGAM and ITGAL were excluded from the candidates. ICAM1 was used for further investigation in the current study. We performed cellular component and subcellular location analysis of nine predicted ICAM2 interacting proteins; the results indicated that the physically interacting ICAM2 proteins were primarily distributed in the plasma membrane (Supplementary Fig. S7B). The results of IF indicated that ICAM1 was primarily detected at the plasma membrane in the choroid plexus epithelial cells (Supplementary Fig. S7C). To investigate whether ICAM2 protein can interact with ICAM1 of choroid plexus cells, the plasmid of recombinant ICAM2 containing a His tag on the C-terminal region was transiently transfected into the HEK293T or MDA-MB-231 and the expression of ICAM2 was examined by IF assay (Supplementary Fig. S7D). The membrane form of ICAM2 proteins, with a molecular weight of 66 KDa, was purified by Ni-beads and imidazole elution (Supplementary Fig. S7E–F). Further, the results of the pull-down assay using ICAM2-His(6x) showed that purified ICAM2-His(6x) can physically interact with endogenous ICAM1 proteins in the choroid plexus epithelial cells, however, the ITGAL, which is a subunit of LFA-1, cannot be detected in the pull-down lysates (Supplementary Fig. S7G). Together with that the numbers of LeptoM3 cells interacting with choroid plexus epithelial cells were increased in a time-dependent manner compared with MDA-MB-231 cells in the coculture system (Supplementary Fig. S7H) and the IF assay results revealed that ICAM2 colocalized with ICAM1 in the choroid plexus epithelial cells at the interface (Fig. 5E). To examine whether the interaction between ICAM1 and ICAM2 is through direct or indirect binding, the recombinant proteins of GST-ICAM2, were purchased from Abnova, and ICAM1-His(9x) was purified by Ni-beads, respectively (Supplementary Fig. S7I) and subsequentially subjected for investigating the direct protein binding of ICAM1 to ICAM2. The results showed that purified ICAM1-His(9x) proteins can be detected in the GST-ICAM2 pull-down assay but not in the GSH-beads (Fig. 5F) indicated the direct binging of ICAM1 to ICAM2. This conclusion was supported by the GST-ICAM2 proteins can also be detected in the purified ICAM1-His(9x) pull-down assay but not in the Ni-beads (Fig. 5G). These results demonstrated that ICAM2-mediated LM in TNBC through ICAM2-ICAM1 direct interaction but not LFA-1.

### Neutralizing ICAM2 abolishes the leptomeningeal metastasis in vitro and in vivo

**Fig. 6:**
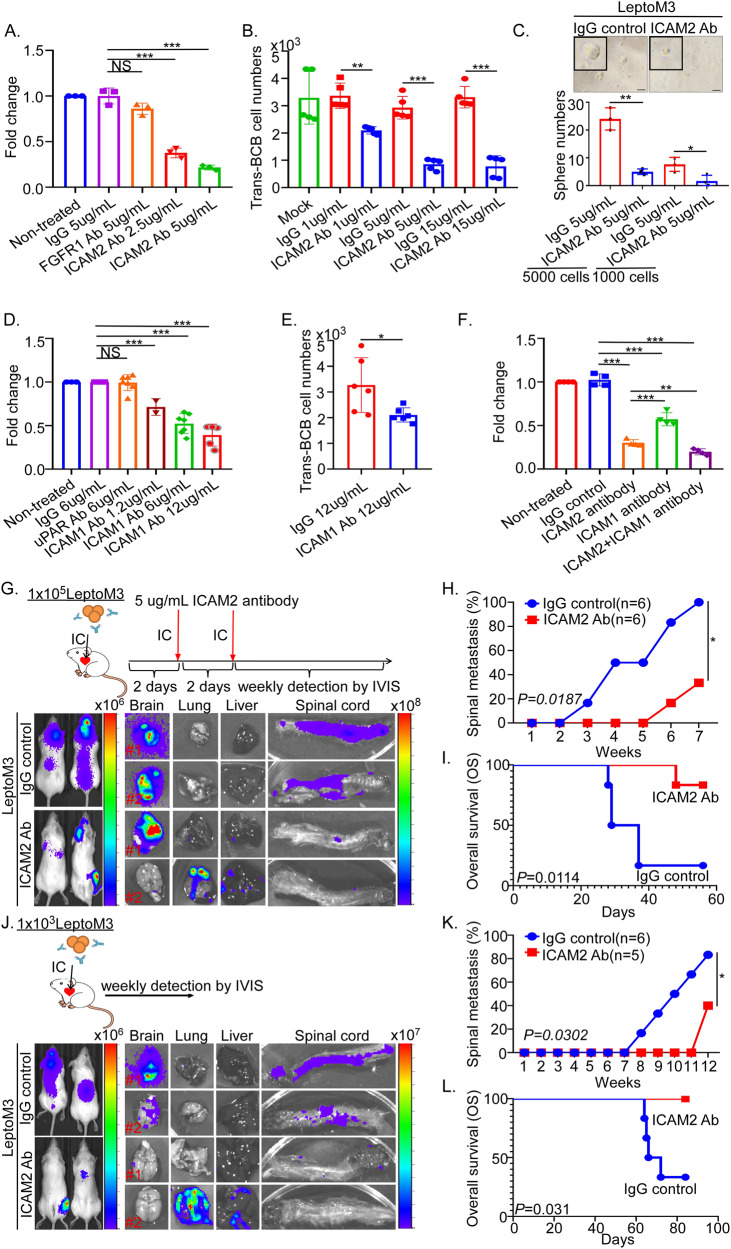
ICAM2 or ICAM1 neutralizing antibodies attenuate the leptomeningeal metastasis in vitro and in vivo. **A** LeptoM3 cells pre-incubated with IgG control antibody (5 µg/mL), FGFR1 antibody (5 µg/mL), and ICAM2 neutralizing antibody (2.5 µg/mL and 5 µg/mL) for 30 mins were subjected to test BCB adhesion ability. **B** LeptoM3 cells pre-incubated with different doses of IgG control and ICAM2 neutralizing antibody for 30 mins were subjected to test Trans-BCB migration ability. **C** 1000 or 5000 LeptoM3 cells treated with IgG and ICAM2 antibody were subjected to sphere forming assay. Scale bar = 200 mm. **D** The in vitro artificial BCB was pre-incubated with IgG control (6 µg/mL), uPAR antibody (6 µg/mL), and ICAM1 neutralizing antibody (1.2 µg/mL, 6 µg/mL and 12 µg/mL) for 30 mins. Following, BCB adhesion ability of LeptoM3 were tested. **E** BCB barrier was pre-incubated with 12 µg/mL of IgG control and ICAM1 neutralizing antibody for 30 mins. Trans-BCB migration ability of LeptoM3 were investigated. **F** BCB adhesion ability was investigated in different groups including (i) LeptoM3 cells and artificial BCB were treated with IgG control; (ii) LeptoM3 cells were treated with ICAM2 neutralizing antibody (5 µg/mL); (iii) artificial BCB was treated with ICAM1 neutralizing antibody (12 µg/mL); (iv) LeptoM3 cells were treated with ICAM2 neutralizing antibody (5 µg/mL) and artificial BCB was treated with ICAM1 neutralizing antibody (12 µg/mL). **G** Metastatic lesions were quantitatively analyzed using IVIS in IgG control (*n* = 6) or ICAM2 antibody (*n* = 6) treated group after 7 weeks of IC injection in vivo and ex vivo. **H** The percentage of leptomeningeal metastasis in IgG control (*n* = 6) or ICAM2 antibody (*n* = 6) treated mice was evaluated (*P* = *0.0187*). **I** Kaplan–Meier analysis revealed the overall survival in xenograft mice. The Log-rank test was applied to evaluate the significance of overall survival rate between the IgG control-treated group and ICAM2 antibodies treated group. **J** Metastatic lesions were quantitatively analyzed using IVIS in IgG control (*n* = 6) or ICAM2 antibody (*n* = 5) treated group after 10 weeks of IC injection in vivo and ex vivo. **K** The percentage of leptomeningeal metastasis in IgG control (*n* = 6) or ICAM2 antibody (*n* = 5) treated mice was evaluated (*P* = *0.0302*). **L** Kaplan–Meier analysis revealed the overall survival in xenograft mice. The Log-rank test was applied to evaluate the significance of overall survival rate between the IgG control-treated group and ICAM2 antibodies treated group. (*P < 0.05; **P < 0.01; ***P < 0.001; NS no significant difference).

Blocking the ICAM2-ICAM1 interaction was proposed to abolish the ICAM2-mediated LM. To investigate whether blockade of ICAM2 and interruption of ICAM2 and ICAM1 interaction can act as potential therapeutic and preventive approaches for LM, anti-ICAM2 or anti-ICAM1 neutralizing antibodies were used to treat LeptoM3 cells or choroid plexus epithelial cells in vitro. The cell numbers of BCB adhesion significantly decreased in LeptoM3 cells treated with 2.5 µg/mL and 5.0 µg/mL anti-ICAM2 neutralizing antibodies compared with the IgG control-treated group in a dose-dependent manner (Fig. 6A). The cell numbers of trans-BCB migration notably decreased in the anti-ICAM2 neutralizing antibody-treated group compared with the IgG control group in a dose-dependent manner (Fig. 6B). The sphere numbers significantly decreased in the anti-ICAM2 neutralizing antibody group compared with the IgG control group (Fig. 6C and Supplementary Fig. S8A). The invasive cell numbers significantly decreased in the anti-ICAM2 neutralizing antibody group compared with the IgG control group in a dose-dependent manner (Supplementary Fig. S8B). Furthermore, the cell numbers of BCB adhesion in the choroid plexus monolayer treatment with 1.2, 6.0, and 12.0 µg/mL anti-ICAM1 neutralizing antibodies significantly decreased compared with the IgG control-treated group in a dose-dependent manner (Fig. 6D). BCB pretreatment with an anti-ICAM1 neutralizing antibody significantly decreased the cell numbers of trans-BCB compared with the IgG control-treated group (Fig. 6E). Combining anti-ICAM2 with anti-ICAM1 neutralizing antibody treatment synergistically reduced the BCB adhesion ability of LeptoM3 cells compared with treatment with only anti-ICAM2 or anti-ICAM1 neutralizing antibodies (Fig. 6F). These results indicated that the blockade of the interaction of ICAM2 and ICAM1 attenuated the progression of LM, in addition to attenuating BCB adhesion, trans-BCB, and stemness abilities in vitro.

To investigate the efficacy of treatment with anti-ICAM2 neutralizing antibodies as a therapeutic approach for LM, the proposed murine model mimics patients with BC who have not undergone surgery. The mice were IC injected with 1 × 10^5^ LeptoM3 cells, which were pre-incubated with 5 µg/mL IgG control or ICAM2 antibodies. Then, antibodies were IC administered every 2 days. The LeptoM3 cells treated with 5 µg/mL IgG control or ICAM2 antibody could not change the protein levels of ICAM2 (Supplementary Fig. S8C). After 7 weeks of IC injection, the results indicated that IgG control-treated group had mice with the colonization of the spinal cord. However, the ICAM2 antibody-treated group attenuates the colonization of the spinal cord in vivo and ex vivo (Fig. 6G and Supplementary Fig. S8D). Early progression of LM in the mice injected with LeptoM3 cells and treated with IgG control were detected in the third week after injection, whereas progression in the ICAM2 antibody-treated group was not detectable till the sixth week after injection (Fig. 6H). Compared with the IgG control-treated group, the survival rate of the mice injected with LeptoM3 cells following treatment with ICAM2 antibody was significantly decreased (Fig. 6I, *p* = 0.0114, median survival = 29.8 days in IgG control-treated group). We investigated whether pretreatment with anti-ICAM2 neutralizing antibodies in 1 × 10^3^ LeptoM3 cells to mimic blocking the circulating tumor cells after surgical resection could be applied as preventive care for LM in TNBC. The mice were IC injected with 1 × 10^3^ LeptoM3 cells, which were pre-incubated with 5 µg/mL IgG control or ICAM2 antibody. After 12 weeks of IC injection, 83.3% mice in the IgG control-treated group had LM; however, the ICAM2 antibody-pretreated group attenuated colonization in the spinal cord ex vivo and in vivo (Fig. 6J, K and Supplementary Fig. S8E). Figure 6L illustrates that pretreated ICAM2 antibody significantly prolonged the survival rate of the mice compared with IgG control treatment (*p* = 0.031, median survival=65.6 days in IgG control-treated group). These results demonstrated that the blockade of ICAM2 in TNBC cells prevented early LM and indeed can act as a potential therapeutic and preventive target for LM in vivo.

## Discussion

**Fig. 7:**
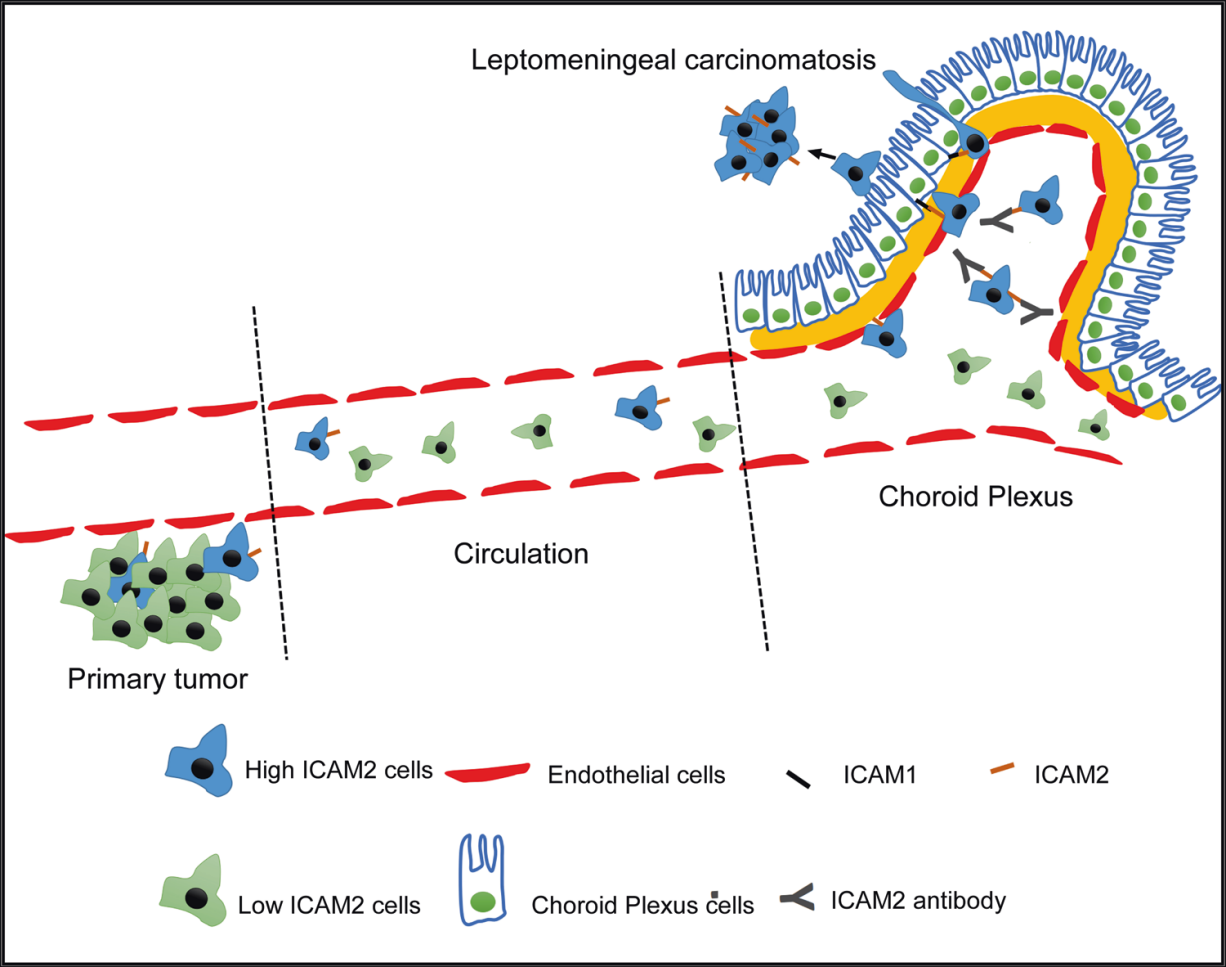
High ICAM2 TNBC cells accumulate on the BCB by ICAM2-ICAM1 interaction to promote the leptomeningeal metastasis. In the circulating system, the high expression of ICAM2 in metastatic TNBC cells helps tumor cells adhere to the Choroid Plexus Epithelial cells through ICAM2-ICAM1 interaction and promotes the Trans-BCB migration to form secondary tumors in the CSF compartment. Blockade of ICAM2 in TNBC cells attenuates the BCB adhesion and Trans-BCB migration of tumor cells.

This study demonstrated that isolated LeptoM3 cells caused specifically early LM and poor survival in vivo (Fig. 1). High expression of ICAM2 and low expression of ICAM1 were correlated with LM in vitro and in vivo (Fig. 2). High ICAM2 in TNBC promoted LM in vivo and enhanced BCB adhesion, trans-BCB migration, and stemness properties in vitro and in vivo (Figs. 3 and 4). High ICAM2 in TNBC cells promoted the specificity of LM through the interactions of ICAM2 and ICAM1 in the choroid plexus epithelial cells (Fig. 5). Moreover, the blockade of ICAM2 by anti-ICAM2 neutralizing antibody abolished the LM and prolonged survival in vivo (Fig. 6G–L). Thus, a primary tumor exhibits heterogenous TNBC cells with high levels of ICAM2. Furthermore, high levels of ICAM2 in TNBC cells enhanced tumor cell adhesion to the choroid plexus epithelial cells of the BCB. ICAM2 also promoted the transmigration of tumor cells across the BCB. High ICAM2 in TNBC cells induced high stemness and initiation abilities, enabling the formation of secondary tumors in the CSF compartment and thus causing leptomeningeal carcinomatosis (Fig. 7).

ICAM2 serves as a tumorigenesis suppressor or promoter and interacts with α-actinin, thereby contributing to an ICAM2-mediated nonmetastatic phenotype in neuroblastoma cells [36, 37]. Downregulation of ICAM2 expression enhanced the radiosensitivity of OSCC cells and the increased apoptotic phenotype through phosphorylation of Akt and the activation of caspase-3, indicating that ICAM2 can serve as an effective radiotherapeutic target for OSCC [38]. In this study, the high levels of ICAM2 promoted leptomeningeal-tropic metastasis in vivo and increased the number of tumor cells adhering to the BCB and trans-BCB migration abilities in vitro (Fig. 3).

Homophilic interaction of ICAM1-ICAM1 directs homotypic tumor cell clustering formation to promote lung specific metastasis in TNBC [39]. In the present study, ICAM1 and ICAM2 levels in CNS-metastasis tropic TNBC cells were evaluated through Western blotting. The results indicated low expression of ICAM1 and ICAM2 compared with parental cells and indicated BrM-831 [40, 41], and BrM3 [42], which have brain metastasis properties. LeptoM3 cells, which contribute to the specificity of LM (Fig. 1), exhibited low expression of ICAM1 and high expression of ICAM2 contributed to the specificity of LM (Fig. 2L–M). Our data suggest that the expression pattern of ICAM1 and ICAM2 determine organ-tropic metastasis in TNBC and that loss of ICAM1, and simultaneously increased ICAM2 expression, is crucial for treating TNBC LM.

Blocking ICAM1 interactions inhibited CTC cluster formation and lung metastasis, thus serving as a novel therapeutic target for lung metastasis of TNBC [39]. In the present study, the results indicated that treatment with anti-ICAM2 neutralizing antibodies attenuated BCB adhesion, trans-BCB migration, and stemness ability in vitro (Fig. 6A–C) and eliminated the early specificity of LM and prolonged the survival rate in vivo (Fig. 6G–L). Our findings indicated that high expression of ICAM2 contributed to the specificity of LM for TNBC cells, suggesting that ICAM2 inhibition may be an effective therapeutic and preventive strategy for LM in TNBC.

## Materials and methods

### Cell lines

Cell lines (HEK293T, 4T1 and MDA-MB-231) were obtained from ATCC (Supplementary Table S1). The HEK293T and MDA-MB-231 cell lines were grown in Dulbecco’s Modified Eagle Medium (DMEM) + 10% cosmic calf serum (CCS) (HyClone, Logan, UT, USA) + 1% penicillin–streptomycin (P/S) (Caisson Labs, Smithfield, UT, USA). The 4T1 cell lines were grown in Dulbecco’s Modified Eagle Medium (DMEM) + 10% fetal bovine serum (FBS) (HyClone, Logan, UT, USA) + 1% penicillin–streptomycin (P/S) (Caisson Labs, Smithfield, UT, USA). The CNS metastasis TNBC cells BrM-831 (brain metastasis) and BrM3 (brain metastasis) were used in this study. The BrM-831 and BrM3 cells are isolated by two rounds of in vivo selection indicating with brain specific metastasis activity. The MDA-MB-231 and CNS metastasis TNBC cells, including BrM-831 and BrM3 were cultured in the DMEM + 10% CCS, and 1% (P/S). All cells were maintained at 37 °C in a humidified atmosphere of 5% CO_2_.

### Statistical analysis

The statistical analysis will perform by using Prism5 and SPSS17 software (New York, NY, USA). Disease-free survival rate and overall survival rate will estimate using Kaplan–Meier methods and compare among age group using log-ranking tests. Cox proportional hazards regression models with univariate and multivariate analyses will summarize with hazard ratios and 95% confidence intervals. Data from all three independent experiments will present as the mean ± SD and analysis by unpaired two-tailed Student’s *t* test. For all tests, *p* < 0.05 defines statistical significance (*p < 0.05, **p < 0.01, ***p < 0.001).

More materials and methods are included in the Supplementary files due to the space limit (Supplementary Materials and Methods).

### Supplementary information

supplementary information

## Data Availability

This published article and supplementary information include all data generated or analyzed in this study. The physically interacting ICAM2 proteins and KEGG pathway and molecular function analysis were analyzed from STRING datasets, which are available at https://string-db.org/. The 932 proteins co-occurring with the tissue choroid plexus are available at the following 10.1093/database/baw100.
