# Supplementary material for: ICAM2 initiates trans-blood-CSF barrier migration and stemness properties in leptomeningeal metastasis of triple-negative breast cancer
Source: Oncogene. 2023 Jul 19;42(39):2919–31. doi: 10.1038/s41388-023-02769-5 (PMC10516748; doi:10.1038/s41388-023-02769-5)
Supplement: Supplementary file 1 — supplementary information [file 41388_2023_2769_MOESM1_ESM.pdf]

Supplementary information

Supplementary Materials and Methods

1. *In vivo* selection

All animal experiment protocols were approved by the Institutional Animal Care and Use Committee in National Cheng Kung University (IACUC NO.:107198, 110113). The NOD/SCID female age-matched mice aged 4–6 weeks were obtained from Laboratory Animal Center (National Cheng Kung University) and randomly used for study. 3D Ultrasound was used to locate the heart of NOD/SCID female mice. MDA-MB-231 carried luciferase was intracardiac (IC) injected into the heart of NOD/SCID female mice. The metastatic tumor cells were monitored by IVIS weekly. After metastatic spinal cord metastasis occurrence, the spinal cord tissues were collected and digested by 1X trypsin, following cells were cultured in the culture plates called LeptoM1. This process would be conducted third round to acquire the LeptoM2 and LeptoM3. The LeptoM1, LeptoM2, and LeptoM3 were cultured in DMEM + 10% CCS + 1% P/S and maintained at 37 °C in a humidified atmosphere of 5% CO2.

2. Stable cell establishment

The two independent shRNA plasmids for ICAM2 knockdown were transfected into the LeptoM3 cells and stable knockdown cells were isolated by puromycin

selection at 48 hours after transfection to establish downregulation of ICAM2 LeptoM3 cells. ICAM2 overexpressing MDA-MB-231 cells was isolated and enriched through flow cytometry by Rabbit anti-ICAM2 monoclonal (#13355, Cell Signaling) antibody. Stable downregulation of ICAM2 LeptoM3 cells and ICAM2 overexpressing MDA-MB-231 cells were cultured in the DMEM + 10% CCS, and 1% (P/S). All cells were maintained at 37 °C in a humidified atmosphere of 5% CO2.

3. H&E staining

The slides were incubated at 65°C for 1 hour and then immersed in xylene three times (15 minutes/each) to remove the paraffin. Let slides go through graded ethanol, 100% ethanol twice, 95% ethanol twice, 75% ethanol twice, and 1X PBS in the end. 5 minutes for each concentration. After the sections were deparaffinized and rehydrated, slides were rinsed with distilled water twice. The sections were then counterstained with hematoxylin for 1 minutes and rinsed the slide in tap water for 10 minutes. Next, the sections were stained with eosin for 30s and rinsed the slide in 95% ethanol for 2 minutes. The sections were dehydrated in 75% ethanol twice, 95% ethanol twice, and 100% ethanol twice for 2 minutes for each concentration, followed by being immersed in xylene for 5 minutes three times.

4. Immunohistochemistry (IHC)

Metastatic lesions from mice samples will be dewaxed and rehydrated. Antigen retrieval will then be done by incubating the slides in 10 mM/L citric buffer (pH6.0) and microwaved for 20 minutes. After blocking, the slides will be incubated with primary antibodies against human mitochondria (ab92824, abcam) (1:10,000 dilution), ICAM2 (#13355, Cell Signaling) (1:100 dilution) and ICAM1 (A19300, ABclonal) (1:100 dilution) followed by biotin-conjugated secondary antibody, polymer-HRP and diaminobenzidine tetrahydroxychloride (DAB) solution (DAB, Sigma).

5. Immunofluorescence (IF) staining

The purity of Choroid Plexus Epithelial cells was evaluated and IF staining was performed by primary antibodies including the anti-ICAM1 polyclonal (#MA5-41137**,** ThermoFisher), anti-ICAM2 monoclonal **(**#14-1029-8, ThermoFisher), anti-Claudin-1 (SC-166338, Santacruz), anti-ITGAL (GTX11344, GeneTex), and anti-ITGAM (GTX113089, GeneTex) antibodies incubated at 4℃ overnight. Following washing with 1X PBS. Secondary antibody conjugated with fluorescence dye was used to hybrid with primary antibodies for fluorescence detection.

The LeptoM3 and Choroid Plexus Epithelial cells were co-culture at a density of 2×10^4 in cover slides per well. The IF staining was performed by primary antibodies

including the anti-ICAM1 polyclonal (#MA5-41137**,** ThermoFisher) and anti-ICAM2 monoclonal **(**#14-1029-8, ThermoFisher) antibodies incubated at 4℃ overnight. Following washing with 1X PBS. Secondary antibody conjugated with fluorescence dye was used to hybrid with primary antibodies for fluorescence detection.

The LeptoM3, two independent ICAM2 shRNA knock-down (sh#1 and sh#4) LeptoM3, BrM3, MDA-MB-231, ICAM2 overexpressing MDA-MB-231, and Choroid Plexus Epithelial cells were seeded at a density of 1×10^5 in cover slides per well. The IF staining was performed by primary antibodies including the anti-ICAM1 polyclonal (A19300**,** ABclonal) and anti-ICAM2 monoclonal **(**#13355, Cell Signaling) antibodies incubated at 4℃ overnight. Following washing with 1X PBS. Secondary antibody conjugated with fluorescence dye was used to hybrid with primary antibodies for fluorescence detection.

6. BCB adhesion assay

Poly-L-lysine coated wells were seeded with human Choroid Plexus Epithelial cells (#1310, Science Cell) and maintained in a growth medium (#4101, Science Cell). One day later, Cancer cells were seeded on the top of the Choroid Plexus Epithelial cells monolayer and adhesion for 1-10 mins. Adhesive cell numbers were counted by the luciferase activity of cells.

Cancer cells ($2x10^5$ cells/100uL) were preincubated with 5 μg/mL isotype control IgG, 5 μg/mL anti-FGFR-1 (GTX20829, GeneTex) or 2.5 μg/mL and 5 μg/mL Goat anti-ICAM2 polyclonal (AF244, R&D) antibodies at 4°C for 30 mins. Following, cancer cells seeded on the top of the Choroid Plexus Epithelial cells monolayer and adhesion for 10 mins. Adhesive cell numbers were counted by the luciferase activity of cells.

Poly-L-lysine coated wells were seeded with human Choroid Plexus Epithelial cells (#1310, Science Cell) and maintained in a growth medium (#4101, Science Cell). One day later, Choroid Plexus Epithelial cells monolayer was treated with 6 μg/mL isotype control IgG, 5 μg/mL uPAR (GTX20829, GeneTex) or 1.2 μg/mL, 6 μg/mL and 12 μg/mL Rabbit anti-ICAM1 polyclonal (A19300, ABclonal) antibodies at 37°C for 30 mins. Following, antibodies were washing with 1XHBSS three times. Cancer cells ($2x10^5$ cells/100uL) were seeded on the top of the Choroid Plexus Epithelial cells monolayer and adhesion for 10 mins. Adhesive cell numbers were counted by the luciferase activity of cells.

7. Brain slices preparation and brain slices adhesion

The removal of the brain tissue from the mice following subject for the Rodent Brain Matrix (RBM-2000C), which was designed to aid in the free-hand dissection of

the brain with 1.0 mm thickness for each slice. The physiological conditions of the brain slices were maintained in the DMEM/F12 culture medium for the brain slices adhesion study. 1×10^5 MDA-MB-231, BrM3, and LeptoM3 cells were seeded on the brain slices and cell adhesion was tested for 60 mins. Following, 1XHBSS was used to remove the non-adhesive tumor cells. IVIS and IHC staining were used to investigate the adhesive cell numbers and location of adhesive cells in brain slices *ex vivo*.

8. Sphere forming assay

Ultra-low 96 well plates were prepared and the cells were seeded at a density of 1×10^4 per well in the DMEM/F12 supplemented with 1% N2 supplement, 20 ng/mL epidermal growth factor (EGF), and 20 ng/mL basic fibroblast growth factor (bFGF). The plate was incubated at 37℃ in the incubator for 10 days to allow sphere formation, and the sphere was counted by bright field microscopy.

Ultra-low 96 well plates were prepared and the LeptoM3 cells were seeded at a density of 1×10^3 or 5×10^3 per well in the DMEM/F12 supplemented with 1% N2 supplement, 20 ng/mL EGF, and 20 ng/mL bFGF. Following, 5 μg/mL isotype control IgG or Goat anti-ICAM2 polyclonal (AF244, R&D) antibodies were treated in cultured medium every two days. The plate was incubated at 37℃ in the incubator for 10 days to allow sphere formation.

9. Trans-BCB migration assay

Poly-L-lysine coated 6.5 mm transwell supports (Corning Costar) with 3.0mm pore size were seeded with human Choroid Plexus Epithelial cells (#1310, Science Cell) and maintained in a growth medium (#4101, Science Cell). Three days later, the permeability of the barriers was tested with sodium fluorescein dye (518-47-8, Merck) (0.01%) diluted with a culture medium and incubated for 15 and 30 minutes at 37℃. Medium in the bottom chamber was collected, and absorbance was measured by Elisa reader, the barrier was deemed ready for assays. Cancer cells were seeded on the top of the chamber, were coated with human Choroid Plexus Epithelial cells for 3 days, and cultured at 37℃ for 24 hours. The trans-migrated cells were attracted by DMEM+1% CCS and luciferase activity of cells counted migrated cell numbers.

$1x10^5$ Cancer cells pre-incubated with different doses of IgG control (1 μg/mL, 5 μg/mL, and 15 μg/mL) and ICAM2 antibody (AF244, R&D) (1 μg/mL, 5 μg/mL, and 15 μg/mL) for 30 mins were subjected to test Trans-BCB migration ability. The trans-migrated cells were attracted by DMEM+1% CCS and luciferase activity of cells counted migrated cell numbers.

10. Invasion assays

Trans-well (8um pore size, BD Biosciences) was performed on invasion assay. Trans-wells were placed in 24 well plates and each well filled with 1mL of DMEM medium containing 1% CCS and 1% P/S. For invasion assay, each top of chamber was coated the Matrigel (1mg/mL, BD Biosciences) for 3 hours at 37°C. Cancer cells were seeded on the top of chamber and cultured at 37°C for 18 hours. subsequently, top of chamber was removed from the plate and washed with 1X HBSS three times. Lower side of the filter membrane was fixed using methanol for 20 minutes and stained with 10% Giemsa solution (Sigma-Aldrich, St. Louis, MO) for 10 minutes. Finally, the Lower side of the filter membrane was washed with distilled water three times and cells were counted in bright-field microscopy.

11. Gel assisted digestion

The protein solutions were mixed with SDS sample buffer and eluted at 95°C for 10 min. The proteins were loaded and analyzed by 10% SDS-PAGE (1 cm). The excised gel was first de-stained, and then reduced with 10 mM dithiothreitol (DTT, Merck) at 60°C for 45 min, followed by cysteine-blocking with 55 mM iodoacetamide (IAM, Sigma) at 25°C for 30 min. Samples were digested with sequencing-grade modified porcine trypsin (Promega) at 37°C for 16 hours. The peptides were then extracted from gel, dried by vacuum centrifugation, and reconstituted with 0.5% Formic acid before

analyzing.

12. Protein identification.

The data analysis was carried out using Proteome Discoverer software (version 1.4, Thermo Fisher Scientific). The MS/MS spectra were searched against the SwissProt database using the Mascot search engine (Matrix Science, London, UK; version 2.5). For peptide identification, 10 ppm mass tolerance was permitted for intact peptide masses, and 0.5 Da for CID fragment ions with allowance for two missed cleavages made from the trypsin digestion: oxidized methionine and acetyl (protein N-terminal) as variable modifications; carbamidomethyl (cysteine) as static modification. Peptide-spectrum match (PSM) were then filtered based on high confidence and Mascot search engine rank 1 of peptide identification to ensure an overall false discovery rate below 0.01. Proteins with single peptide hit were kept.

13. Extraction of Membrane and Cytosolic fractions

The protocol is according to the Mem-PER™ Plus Membrane Protein Extraction Kit (#89842, Thermo Fisher). Resuspend 5×10^6 cells in the growth media by scraping the cells off the surface of the plate with a cell scraper. Centrifuge harvested cell suspension at $300 \times g$ for 5 minutes. Wash cell pellet with 3mL of Cell Wash Solution

(included in the kit) and centrifuge. Carefully remove and discard the supernatant. Resuspend the cells in 1.5mL of Cell Wash Solution and centrifuge to discard the supernatant. Add 0.75mL of Permeabilization Buffer (included in the kit) to the cell pellet. Vortex briefly to obtain a homogeneous cell suspension. Incubate for 10 minutes at 4°C with constant mixing. Centrifuge permeabilized cells for 15 minutes at 16,000 × g. Carefully remove the supernatant containing cytosolic proteins and transfer it to a new tube. Add 0.5mL of Solubilization Buffer to the pellet and resuspend by pipetting up and down. Incubate tubes at 4°C for 30 minutes with constant mixing. Centrifuge tubes at 16,000 × g for 15 minutes at 4°C. Transfer supernatant containing solubilized membrane and membrane-associated proteins to a new tube. Proceed to the downstream application. Immediately use cytosolic and membrane fractions stored on ice or store aliquots at -80°C for future use.

14. LC-MS/MS analysis

The digested peptides were diluted in HPLC buffer A (0.1% formic acid) and loaded onto a reverse-phase column (Zorbax 300SB-C18, 0.3 × 5 mm; Agilent Technologies). The desalted peptides were then separated on a homemade column (Waters BEH 1.7 μm, 100 μm I.D. × 10 cm with a 15 μm tip) using a multi-step gradient of HPLC buffer B (99.9% acetonitrile/0.1% formic acid) for 70 minutes with a flow

rate of 0.3 µl/min. The LC apparatus was coupled with a 2D linear ion trap mass spectrometer (Orbitrap Elite ETD; Thermo Fisher) operated using Xcalibur 2.2 software (Thermo Fisher). The full-scan MS was performed in the Orbitrap over a range of 400 to 2,000 Da and a resolution of 120,000 at m/z 400. Internal calibration was performed using the ion signal protonated dodecamethylcyclohexasiloxane ion at m/z 536.165365 as lock mass. The 20 data-dependent MS/MS scan events were followed by one MS scan for the 20 most abundant precursor ions in the preview MS scan. The m/z values selected for MS/MS were dynamically excluded for 40 seconds with a relative mass window of 15 ppm. The electrospray voltage was set to 2.0 kV, and the temperature of the capillary was set to 200°C. MS and MS/MS automatic gain control were set to 1,000 ms (full scan) and 200 ms (MS/MS), or 3 × 106 ions (full scan) and 3,000 ions (MS/MS) for maximum accumulated time or ions, respectively. All datasets will be publicly available for querying and downloading through the GEO database when this paper is accepted.

15. Flow cytometry

The 5×10^5 MDA-MB-231, LeptoM3, and BCB adhesion cells were washed with 1X PBS and were incubated with conjugated antibodies including Mouse-anti-CD133 Alexa488 (130-113-670, Miltenyi Biotec), Mouse-anti-CD24 PE (#555427, BD

Bioscience), Rabbit-anti-EPCAM Alexa488 (GTX636998, GeneTex), and Mouse-anti-CD44 APC (#5599425, BD Bioscience) or isotype control IgG (Upstate Biotechnology) at 4°C for 30 minutes. Following washing, with 1X PBS three times and CD133, CD24, EPCAM, and CD44 positive cell population was detected by BD FACScaliber flow cytometry (BD Biosciences).

16. Purification of ICAM2-His and ICAM1-His proteins

The $1x10^7$ HEK293T cells were transiently transfected with ICAM2-bio-His plasmid (#51758, Addgene) or pD649-Hasp-ICAM1-COMP5AP-AviTag-9xHis plasmid (#157432, Addgene) at 37°C for 4 hours. Following washing, with 1X PBS three times, and extraction of Membrane fraction by the Mem-PER™ Plus Membrane Protein Extraction Kit (#89842, Thermo Fisher). The membrane fraction containing the membrane form of ICAM2-His(6x) or ICAM1-His(9x) was incubated with the appropriate volume of Ni-beads (#30210, QIAGEN) at 4°C for 1 hour. Following, washing the Ni-beads conjugated ICAM2-His(6x) or ICAM1-His(9x) with 10mM imidazole two times. Finally, the 500mM imidazole will be used to eluate the ICAM2-His(6x) or ICAM1-His(9x) proteins from Ni-beads for further usage.

17. Pull down assay

Purified ICAM2-His(6x) proteins were used to incubate with membrane fractions of Choroid Plexus epithelial cells at 4°C overnight. Following, a mixture or only membrane fractions of Choroid Plexus epithelial cells were incubated with Ni-beads (#30210, QIAGEN) at 4°C for 1 hour. Following, washing the mixture with 10mM imidazole two times. Finally, the 500mM imidazole will be used to eluate the ICAM2-His(6x) proteins and their interacting partners from Ni-beads for further investigation.

GST-ICAM2 proteins (H00003384-P01, Abnova) were used to incubate with ICAM1-His (9x), which was preincubated with Ni-beads (#30210, QIAGEN), or Ni-beads only as control at 4°C for 2 hours. In contrast, ICAM1-His (9x) proteins were used to incubate with GST-ICAM2, which was preincubated with GSH-beads (#635607, Clontech), or GSH-beads only as control at 4°C for 2 hours. Finally, pull down lysates were subjected to western blotting analysis.

18. ***Xenograft animal model***

The leptomeningeal metastasis mice model of NOD/SCID female age-matched mice aged 4–6 weeks were obtained from Laboratory Animal Center (National Cheng Kung University) and randomly used for xenograft studies. TNBCs were injected into the left ventricle of the heart of NOD/SCID mice through intracardiac injection. The tumor metastasis and growth were detected by IVIS measurement weekly. After 4

weeks of IC injection, the IVIS combined with Micro-CT was used to detect the specificity of spinal cord metastasis in mice (IVIS signal indicating the distribution of tumor cells and Micro-CT image indicating the location of signal). Animals were sacrificed at 7-10 weeks and then the organ can be collected for *ex vivo* detection.

The MDA-MB-231, BrM3, and LeptoM3 cells were IC injected into the NOD/SCID mice and metastasis was detected by the IVIS system every week. All criteria of euthanasia were in accordance with animal welfare regulations. First, according to the spirit of 3R, all the groups were sacrificed by CO_2 euthanasia at endpoint, because our results demonstrated that the occurrence of leptomeningeal metastasis in mice was detected by IVIS *in vivo* and *ex vivo* at the 7th week after IC injection. Second, we sacrificed mice with severe metastasis including brain, lung, and spinal cord (photon flux signal higher than $1x10^7$) accompanied with mobility, and significant weight loss issues, which is evaluated and informed by a professional veterinarian, by CO_2 euthanasia before the 7 weeks.

For evaluating LM in normal background mice, 1×10^5 or 1×10^4 ICAM2 overexpressing 4T1 cells and vector control cells carries the luciferase were IC injected into the BALB/c mice and distribution of cancer cells were monitored by IVIS every two to three days. According to the spirit of 3R, we will sacrifice mice with sever metastasis including brain, lung, and spinal cord (photon flux signal higher than $1x10^7$)

accompanied with mobility issues by CO_2 Euthanasia at the human endpoint every day. All mice were sacrificed on the humanitarian end point.

For establishing a tumor initiation model, 1×10^5, 1×10^4, and 1×10^3 ICAM2 overexpressing MDA-MB-231/ 50μL HBSS or vector control MDA-MB-231/ 50μL HBSS mixed with 50μL Matrigel (9.7mg/mL, BD Biosciences) were injected into the mammary fat pads of NOD/SCID female mice. Tumor growth was detected using IVIS once a week. The animals were sacrificed at 7–20 weeks. The organs were then collected and subjected to IVIS detection *ex vivo*.

The 1×10^5 LeptoM3 cells, which were preincubated with 5 μg/mL IgG control or ICAM2 antibodies (AF244, R&D), following injecting into the NOD/SCID mice (n=6/each group). Then, 5 μg/mL ICAM2 antibodies (AF244, R&D), which dissociated with 1xHBSS, were IC administered every 2 days following metastasis were monitored by IVIS every week. After 7 weeks, all mice were sacrificed and metastatic lesions in organ were detected by IVIS system. All criteria of euthanasia were in accordance with animal welfare regulations. First, according to the spirit of 3R, all the groups were sacrificed by CO_2 euthanasia at endpoint, because our results demonstrated that the occurrence of leptomeningeal metastasis in mice was detected by IVIS *in vivo* and *ex vivo* at the 7th week after IC injection. Second, we sacrificed mice with severe metastasis including brain, lung, and spinal cord (photon flux signal higher than $1\text{x}10^7$)

accompanied with mobility, and significant weight loss issues, which is evaluated and informed by a professional veterinarian, by CO_2 euthanasia before the 7 weeks.

The 1×10^3 LeptoM3 cells, which were preincubated with 5 μg/mL IgG control or ICAM2 antibodies (AF244, R&D), were injected into the NOD/SCID mice (n=6/IgG control group, n=5/ICAM2 antibodies treated group) following metastasis was monitored by IVIS every week. After 12 weeks, all mice were sacrificed and metastatic lesions in organ were detected by IVIS system. All criteria of euthanasia were in accordance with animal welfare regulations. First, according to the spirit of 3R, all the groups were sacrificed by CO_2 euthanasia at endpoint, because our results demonstrated that the occurrence of leptomeningeal metastasis in mice was detected by IVIS *in vivo* and *ex vivo* at the 12th week after IC injection. Second, we sacrificed mice with severe metastasis including brain, lung, and spinal cord (photon flux signal higher than 1×10^7) accompanied with mobility, and significant weight loss issues, which is evaluated and informed by a professional veterinarian, by CO_2 euthanasia before the 12 weeks.

19. Western Blotting

Cell lysates are loaded on SDS-polyacrylamide gel for electrophoresis and transferred to PVDF membranes. Protein expressions are examined after incubation with primary antibodies (**Supplementary Table S1**) followed by HRP-conjugated

secondary antibodies and detected by X-ray film.

20. QRT-PCR

Total RNA isolation and reverse transcription will conduct using the method described previously. The mRNA of AGRV1, OR8S1, VTN, ITGA2, ICAM2, EMT-related, stemness, drug resistance genes, MMP families, and GAPDH were analysis by qRT-PCR. The primer sequence was listed in **Supplementary Table S2**. The results are normalized to those of the housekeeping gene glyceraldehyde-3-phosphate dehydrogenase (GAPDH).

Supplementary Figure Legends

Supplementary Figure 1. The isolated LeptoM3 cells promote the specificity of spinal cord colonization *in vivo*. A. 1×10^5 MDA-MB-231 cells were injected into the left ventricle of the heart through IC injection, following metastatic brain lesions were collected at 7 weeks after IC injection. IHC detected metastatic tumor cells in xenografted brain tissues. **B.** 1×10^5 MDA-MB-231, BrM3, LeptoM1, LeptoM2 and LeptoM3 cells were injected into the left ventricle of the heart through IC injection. After 3-7 weeks of IC injection, the metastatic lesions were detected in different organs by IVIS system *ex vivo*. **C.** Brain colonization of BrM3 cells can be detected by IVIS

combined with Micro-CT analysis 4 weeks after IC injection. **D.** The decision images of NOD/SCID mice injected with MDA-MB-231, BrM3, and LeptoM3 cells and distribution of cancer cells were detected by IVIS weekly *in vivo*. The median survival days of LeptoM3 were 31.2 days, however, the median survival days of BrM3 or MDA-MB-231 were 36.4 days.

Supplementary Figure 2. *In vitro* artificial BCB system is established and measured by sodium fluorescence tracking dye. A. The model of the *in vitro* artificial BCB establishment and permeability test. **B.** 1×10^5 Choroid Plexus cells were cultured in the trans-well insert for 1 day to form an artificial BCB monolayer. The sodium fluorescence tracking dye (0.01mg/mL) was used to evaluate BCB permeability. The fluorescence intensity represented the integrity of the BCB. **C.** The standard curve of cell numbers was calculated by detection of luciferase activity in various triple-negative breast cancer cells (MDA-MB-231, BrM3, and LeptoM3 cells) (***P<0.001)

Supplementary Figure 3. Leptomeningeal-tropic cells increase the invasive ability. A. Invasion ability analysis was conducted in (1) MDA-MB-231, (2) BrM3, and (3) LeptoM3 cells for 24 hours. **B.** IHC staining was used to detect location of adhesive tumor cells in brain slices (Black arrow indicated tumor cells adhere to pia mater).

(***$P < 0.001$)

Supplementary Figure 4. Leptomeningeal-tropic cells and BCB adhesion cells significantly increase the protein levels of ICAM2. A. The protein levels of ICAM2 and ICAM1 were examined using western blotting. **B.** Silver staining combined with in gel digestion proteomics demonstrated that ICAM2 exist in the LeptoM3 cells. **C.** The sub-cellular location of ICAM1 and ICAM2 were investigated in MDA-MB-231, BrM3, and LeptoM3 cells by IF staining assay. **D.** The protein levels of ICAM2 were detected in LeptoM1, LeptoM2, and LeptoM3 compared with MDA-MB-231 cells by western blot. **E.** The scheme of selective approach for BCB adhesion cells derived from MDA-MB-231. **F.** The protein levels of ICAM2 and ICAM1 were examined in ICAM2 positive MDA-MB-231 cells using western blotting. **G.** The cell morphology of ICAM2 positive MDA-MB-231 and parental MDA-MB-231 cells. **H.** The sub-cellular location of ICAM2 were investigated in ICAM2 positive MDA-MB-231 cells by IF staining assay (White arrow indicated that the membrane location of ICAM2). **I.** BCB adhesion ability of MDA-MB-231 and ICAM2 positive MDA-MB-231 cells was investigated by using *in vitro* artificial BCB. The luciferase activity of cells counted adhesive cell numbers. (**$P < 0.01$; ***$P < 0.001$; NS no significant difference)

Supplementary Figure 5. Down-regulation or Overexpression of ICAM2 in MDA-MB-231 or 4T1 cells attenuated or promoted the early LM *in vivo*.

A. 1×10^5 ICAM2 downregulating or scramble control LeptoM3 cells were IC injected into the heart of NOD/SCID mice, following metastatic cells were monitored by IVIS weekly. **B.** 1×10^5 ICAM2 overexpressing or vector control MDA-MB-231 cells were IC injected into the heart of NOD/SCID mice, following metastatic cells were monitored by IVIS weekly. **C.** $1x10^4$ vector control and ICAM2 overexpressing 4T1 cells were IC injection in BALB/c mice. Quantitative analysis of vector control and ICAM2 overexpressing 4T1 cells tracing by IVIS. **D.** 1×10^5 ICAM2 overexpressing or vector control 4T1 cells were IC injected into the heart of BALB/c mice, following metastatic cells were monitored by IVIS weekly. After 12 days, the metastatic lesions on multiple organs were detected by IVIS *ex vivo*. **E.** The metastatic percentage of the different organ metastasis was calculated after 12 days of IC injection with 1×10^5 ICAM2 overexpressing or vector control 4T1 cells in BALB/c mice. **F.** The percentage of leptomeningeal metastasis in ICAM2 overexpressing control (n=4) or vector control (n=4) was evaluated.

Supplementary Figure 6. High levels of ICAM2 in TNBC cells significantly increase the stemness abilities. A. The protein levels of ICAM2, CD44, and CD133

were evaluated using western blotting in ICAM2 overexpressing MDA-MB-231 cells and MDA-MB-231 cells. **B.** The mRNA levels of stemness-related genes including *OCT-4*, *SOX2*, *NANOG*, *NOTCH1*, and *PROM1* were examined using qRT-PCR in ICAM2 overexpressing MDA-MB-231 compared with MDA-MB-231. **C.** The protein levels of ICAM2, CD44, and CD133 were evaluated using western blotting in two independent ICAM2 knock-down LeptoM3 cells and LeptoM3 cells**.** **D.** The table presented the percentage of tumor formation (photon flux signal >$1.7x10^4$) was calculated in NOD/SCID mice orthotopic injected with vector control or ICAM2 overexpressing MDA-MB-231 cells. **E**. The protein levels of ICAM2, CD44, and CD133 were examined using western blotting in (1) MDA-MB-231, (2) BrM3, (3) LeptoM3, (4) BCB adhesion cells, (5) BBB adhesion cells. (**$P < 0.01$; ***$P < 0.001$)

Supplementary Figure 7. ICAM1 specifically express on the plasma membrane of Choroid Plexus cells. A. The protein levels of ICAM1 were examined using western blotting in HUVEC and Choroid Plexus cells. **B.** Cellular Component and Subcellular location analysis were used to analyze involved pathways in 9 ICAM2 interacting candidates. **C.** The sub-cellular location of claudin1, ICAM1, ITGAL, ICAM2, and ITGAM were investigated in Choroid Plexus cells by IF staining assay. **D.** The location of His-ICAM2 in transiently transfected HEK293T and MDA-MB-231 cells was

evaluated using the IF assay. **E.** The ICAM2-His proteins expression of membrane and cytosolic fraction extraction were determined by western blotting with anti-His and anti-ICAM2 antibodies in ICAM2 overexpression plasmid transiently transfected HEK293T cells. **F.** Membrane form of ICAM2-His proteins by Ni-beads binding and eluting by imidazole was confirmed by western blotting. **G.** Pull-down assay detected binding between purified ICAM2 protein and endogenous ICAM1 of choroid plexus cells. The pull-down assay was carried out by incubating Nickel beads with or without His-ICAM2. After coprecipitation with membrane fraction of choroid plexus cells, the isolated proteins were analyzed by anti-ICAM2, anti-ITGAL, and anti-ICAM1 by western blotting. **H**. Choroid Plexus epithelial cells interacted with LeptoM3 cells after 8, 16, and 24 hours in the co-culture system. **I.** The purified ICAM2 and ICAM1 proteins were investigated by western blotting. (**$P < 0.01$; ***$P < 0.001$; NS no significant difference)

Supplementary Figure 8. Neutralizing ICAM2 by neutralizing antibodies attenuated the invasive ability *in vitro*. A. The scheme of LeptoM3 cells treated with IgG control or ICAM2 antibody for sphere-forming assay. **B.** $3x10^4$ LeptoM3 cells treated with various dose neutralizing antibodies were subjected for invasive ability test including (i) Mock; (ii) IgG control (1μg/mL) treatment; (iii) ICAM2 antibody

(1μg/mL) treatment; (iv) IgG control (5μg/mL) treatment; (v) ICAM2 antibody (5μg/mL) treatment; (vi) IgG control (15μg/mL) treatment; (vii) ICAM2 antibody (15μg/mL) treatment. **C.** The protein levels of ICAM2 and ICAM1 were investigated in $1x10^5$ LeptoM3 cells treated with IgG control (5μg/mL) or ICAM2 antibody (5μg/mL) by western blot. **D.** Metastatic lesions were quantitatively analyzed using IVIS in IgG control or ICAM2 antibody-treated mice IC injected with 1×10^5 LeptoM3 cells after 7 weeks of IC injection *ex vivo*. **E.** Metastatic lesions were quantitatively analyzed using IVIS in IgG control or ICAM2 antibody-treated mice IC injected with 1×10^3 LeptoM3 cells after 12 weeks of IC injection *ex vivo*. (**$P < 0.01$; ***$P < 0.001$)

Supplementary Table S1

**Western blot Antibodies**		
ICAM2	#13355	Cell Signaling
ICAM1	A19300	ABclonal
Tubulin	T5168	Sigma
GAPDH	Ab8245	abcam
CD133	Ab222782	abcam
CD44	#BBA10	R&D
ITGAM	GTX113089	GeneTex
ITGAL	GTX11344	GeneTex
**IF Antibodies**		
ICAM2	#13355	Cell Signaling
ICAM2	# 14-1029-8	ThermoFisher
ICAM1	A19300	ABclonal
ICAM1	# MA5-41137	ThermoFisher
Hoechest	#33342	ThermoFisher
CD44	#BBA10	R&D

ITGAM	GTX113089	GeneTex
ITGAL	GTX11344	GeneTex
Claudin-1	SC-166338	Santacruz
**IHC Antibodies**		
human mitochondria	ab92824	abcam
ICAM2	#13355	Cell Signaling
ICAM1	A19300	ABclonal

**shRNA clone and ICAM2-His clone (for human genes)**		
ICAM2-bio-His	Plasmid #51758	Addgene
pD649-Hasp-ICAM1-COMP5AP-AviTag-9xHis plasmid	Plasmid #157432	Addgene
shRNA clone for ICAM2 #1	TRCN0000057821 NM_000873	Academia Sinica (RNAi core)
shRNA clone for ICAM2 #4	TRCN0000057820 NM_000873	Academia Sinica (RNAi core)

**Cell Lines**		
MDA-MB-231	ATCC	ATCC® HTB-26™
4T1	ATCC	ATCC® CRL-2539™
HEK293T	ATCC	ATCC® CRL-11268™

Supplementary Table S2

qRT-PCR primer list	
Primer	Sequences (5'-3')
GAPDH-Forward	TGAAGGTCGGAGTCAACGGATT
GAPDH-Reverse	CCTGGAAGATGGTGATGGGATT
CDH1-Forward	GAAAGCGGCTGATACTGACC
CDH1-Reverse	CGTACATGTCAGCCGCTTC
CDH2-Forward	TGTTTGACTATGAAGGCAGTGG
CDH2-Reverse	TCAGTCATCACCTCCACCAT
ZO-1-Forward	GTGTTGTGGATACCTTGT

ZO-1-Reverse	GATGATGCCTCGTTCTAC
VIM-Forward	AGGCAAAGCAGGAGTCCACTGA
VIM-Reverse	ATCTGGCGTTCCAGGGACTCAT
FN-Forward	ACCTGTACACCTTGAATGACA
FN-Reverse	TGATACCAGCAAGGAATTGGG
ZEB1-Forward	TTCAAACCCATAGTGGTTGCT
ZEB1-Reverse	TGGGAGATACCAAACCAACTG
ZEB2-Forward	AAATGGACTGCAAGGCTGAA
ZEB2-Reverse	AGGTTGAGAGCATGGATCCT
SNAL1-Forward	ACCACTATGCCGCGCTCTT
SNAL1-Reverse	GGTCGTAGGGCTGCTGGAA
SLUG-Forward	ATCTGCGGCAAGGCGTTTTCCA
SLUG-Reverse	GAGCCCTCAGATTTGACCTGTC
CTNNB1-Forward	ACAACTGTTTTGAAAATCCA
CTNNB1-Reverse	CGAGTCATTGCATACTGTCC
SOX2-Forward	AAATGGGAGGGGTGCAAAAGAGGAG
SOX2-Reverse	CAGCTGTCATTTGCTGTGGGTGATG
OCT4-Forward	CTTGCTGCAGAAGTGGGTGGAGGAA
OCT4-Reverse	CTGCAGTGTGGGTTTCGGGCA
NANOG-Forward	AATACCTCAGCCTCCAGCAGATG
NANOG-Reverse	TGCGTCACACCATTGCTATTCTTC
NES-Forward	TGGCTCAGAGGAAGAGTCTGA
NES-Reverse	TCCCCCATTTACATGCTGTGA
PROM1-Forward	AGTGGCATCGTGCAAACCTG
PROM1-Reverse	CTCCGAATCCATTCGACGATAGTA
CD44-Forward	AAGACATCTACCCCAGCAAC
CD44-Reverse	TTTGCTCCACCTTCTTGACTCC
ABCG2-Forward	TCATCAGCCTCGATATTCCATCT
ABCG2-Reverse	GGCCCGTGGAACATAAGTCTT
ABCB1-Forward	AAATTGGCTTGACAAGTTGTATATGG
ABCB1-Reverse	CACCAGCATCATGAGAGGAAGTC
MMP9-Forward	AGACGGGTATCCCTTCGACG
MMP9-Reverse	AAACCGAGTTGGAACCACGAC
MMP1-Forward	AGCTAGCTCAGGATGACATTGATG
MMP1-Reverse	GCCGATGGGCTGGACAG
AGRV1-Forward	GAAGGCCAGTTACACTGTGG
AGRV1-Reverse	TATCCTCCAGAGGACGGGAA

OR8S1-Forward	CATCTGCCGCCCACTACTTT
OR8S1-Reverse	GATGAGTGCGTCCAGAAAGC
VTN-Forward	CCCAGTCCAAAGGGAATCCT
VTN-Reverse	AGAGGGAACCGTTCTTGAGG
ITGA2-Forward	ATTTGGAACGGGACTTTCGC
ITGA2-Reverse	TCCTGTTGGTACTTCGGCTT
ICAM2-Forward	GGTTACAGGACCCTGACTGT
ICAM2-Reverse	ACTTCAGGCTGGTTACAGGT

Supplementary Table S3

KEGG analysis

#term ID	Term description	Gene cou	Background gene cou	Strength	FDR	Matching proteins
hsa04810	Regulation of actin cytoskeleton	6	209	1.75	1.11E-07	ITGAL,EZR,SRC,ITGB2,RDX,ITGAM
hsa04670	Leukocyte transendothelial migratio	5	109	1.95	2.54E-07	ICAM1,ITGAL,EZR,ITGB2,ITGAM
hsa04514	Cell adhesion molecules	5	137	1.85	5.13E-07	ICAM1,ITGAL,ITGB2,ICAM2,ITGAM
hsa05152	Tuberculosis	5	168	1.77	1.04E-06	CD209,CLEC4M,SRC,ITGB2,ITGAM
hsa05150	Staphylococcus aureus infection	4	86	1.96	5.77E-06	ICAM1,ITGAL,ITGB2,ITGAM
hsa04650	Natural killer cell mediated cytotoxi	4	121	1.81	1.81E-05	ICAM1,ITGAL,ITGB2,ICAM2
hsa04145	Phagosome	4	142	1.74	2.89E-05	CD209,CLEC4M,ITGB2,ITGAM
hsa04015	Rap1 signaling pathway	4	202	1.59	8.89E-05	ITGAL,SRC,ITGB2,ITGAM
hsa05144	Malaria	3	46	2.11	7.34E-05	ICAM1,ITGAL,ITGB2
hsa05416	Viral myocarditis	3	55	2.03	9.81E-05	ICAM1,ITGAL,ITGB2
hsa05323	Rheumatoid arthritis	3	85	1.84	0.00031	ICAM1,ITGAL,ITGB2
hsa04625	C-type lectin receptor signaling path	3	102	1.76	0.00049	CD209,CLEC4M,SRC
hsa04530	Tight junction	3	156	1.58	0.0016	EZR,SRC,RDX
hsa05205	Proteoglycans in cancer	3	196	1.48	0.0028	EZR,SRC,RDX
hsa05166	Human T-cell leukemia virus 1 infec	3	211	1.44	0.0033	ICAM1,ITGAL,ITGB2
hsa05134	Legionellosis	2	55	1.85	0.0078	ITGB2,ITGAM
hsa05140	Leishmaniasis	2	70	1.75	0.0116	ITGB2,ITGAM
hsa05133	Pertussis	2	74	1.72	0.0122	ITGB2,ITGAM
hsa04610	Complement and coagulation cascad	2	82	1.68	0.0142	ITGB2,ITGAM
hsa05146	Amoebiasis	2	100	1.59	0.0198	ITGB2,ITGAM
hsa05418	Fluid shear stress and atherosclerosi	2	130	1.48	0.0314	ICAM1,SRC
hsa05162	Measles	2	138	1.45	0.0336	CD209,CLEC4M
hsa05206	MicroRNAs in cancer	2	160	1.39	0.0428	EZR,RDX

Supplementary Table S4

Gene Ontology (Molecular Function)

#term ID	Term description	Gene count	Background gene cou	Strength	FDR	Matching proteins
GO:0050839	Cell adhesion molecule binding	8	538	1.46	4.83E-08	ICAM1,ITGAL,EZR,SRC,ITGB2,RDX,ICAM2,ITGAM
GO:0044877	Protein-containing complex bindi	7	1216	1.05	0.0003	ICAM1,ITGAL,EZR,SRC,ITGB2,ICAM2,ITGAM
GO:0005178	Integrin binding	5	147	1.82	6.96E-06	ICAM1,SRC,ITGB2,ICAM2,ITGAM
GO:0042277	Peptide binding	4	299	1.42	0.0044	CD209,CLEC4M,ITGB2,ITGAM
GO:0030369	ICAM-3 receptor activity	3	3	3.29	3.10E-06	CLEC4M,ITGAL,ITGB2
GO:0001618	Virus receptor activity	3	74	1.9	0.0037	ICAM1,CD209,CLEC4M
GO:0051117	ATPase binding	3	89	1.82	0.0044	EZR,SRC,RDX
GO:0001851	Complement component c3b binc	2	5	2.89	0.0032	ITGB2,ITGAM
GO:0046790	Virion binding	2	11	2.55	0.0059	CD209,CLEC4M
GO:0005537	Mannose binding	2	20	2.29	0.0134	CD209,CLEC4M
GO:0042605	Peptide antigen binding	2	22	2.25	0.0139	CD209,CLEC4M

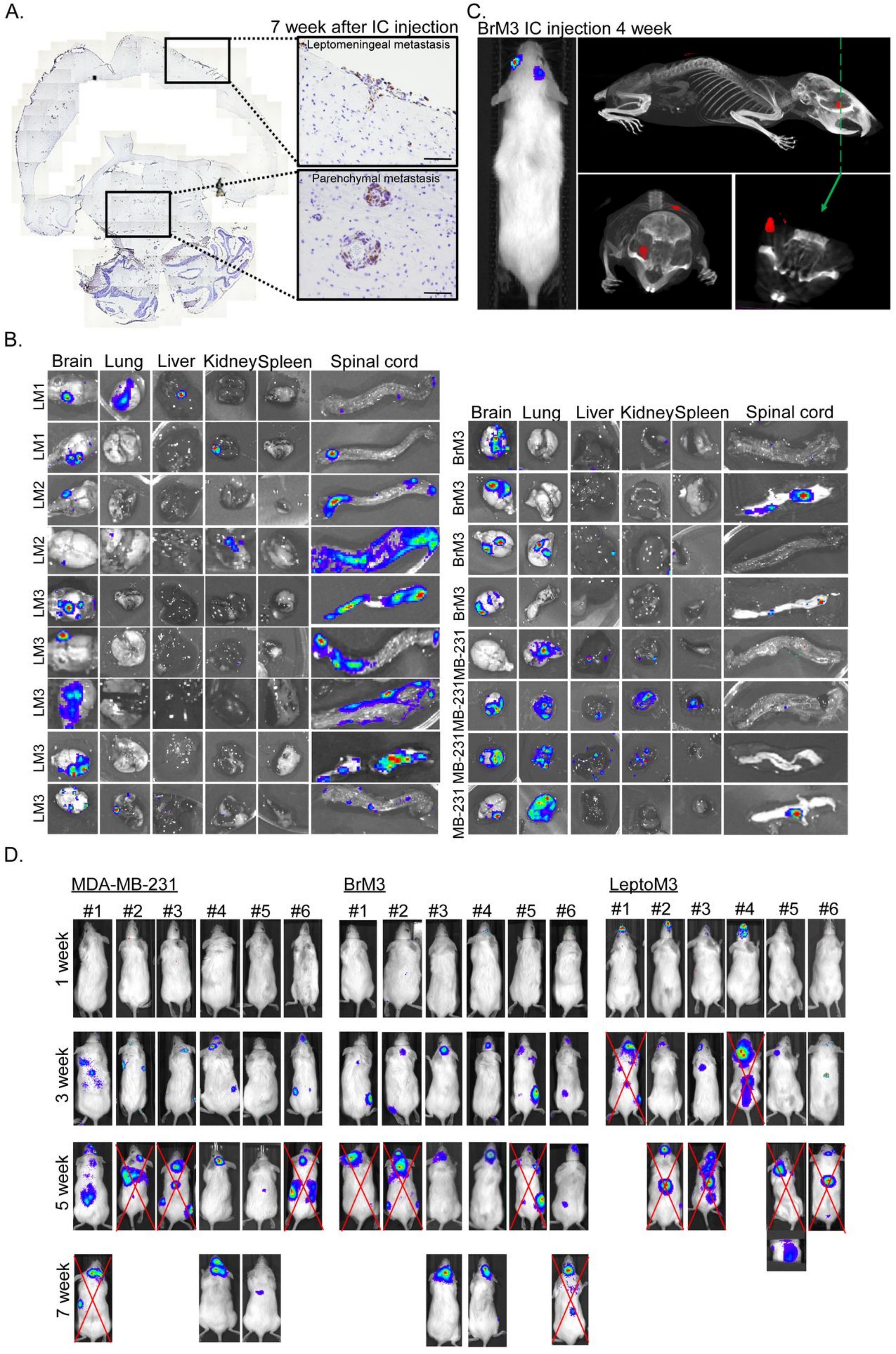

Figure S1 Pan et al.

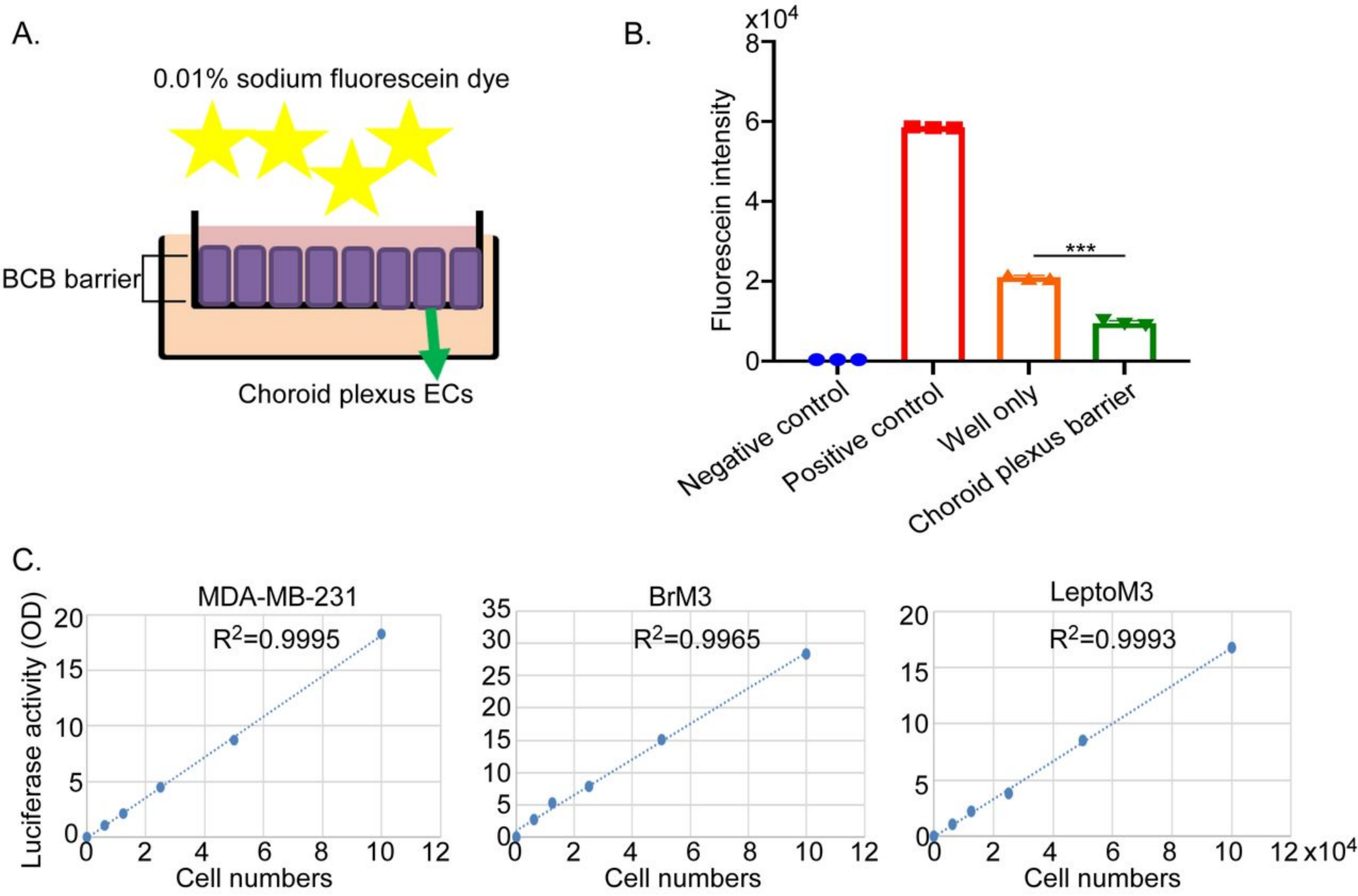

Figure S2 Pan et al.

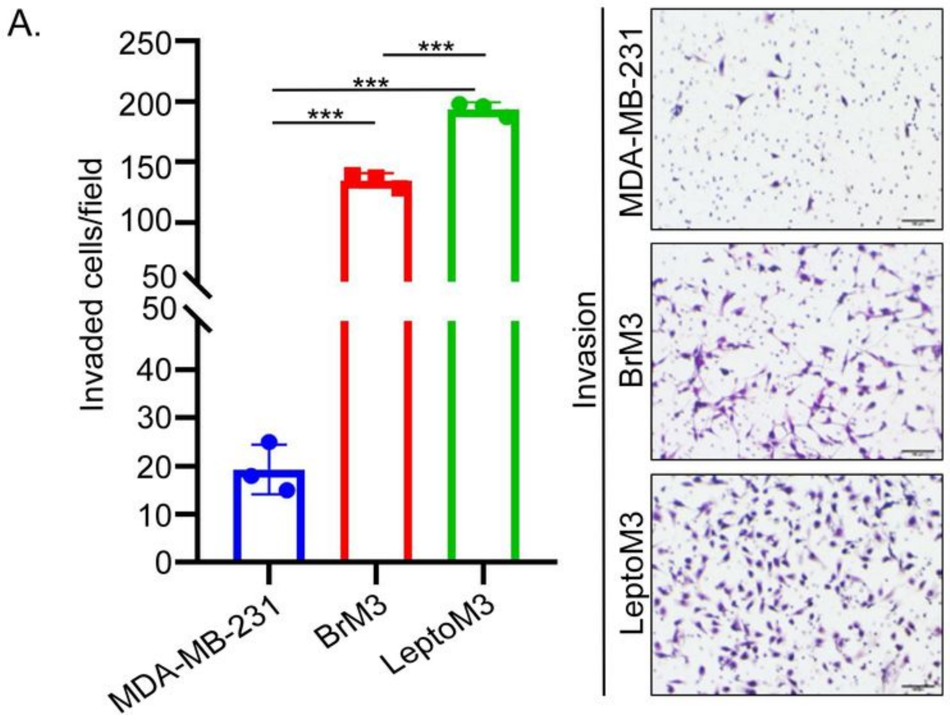

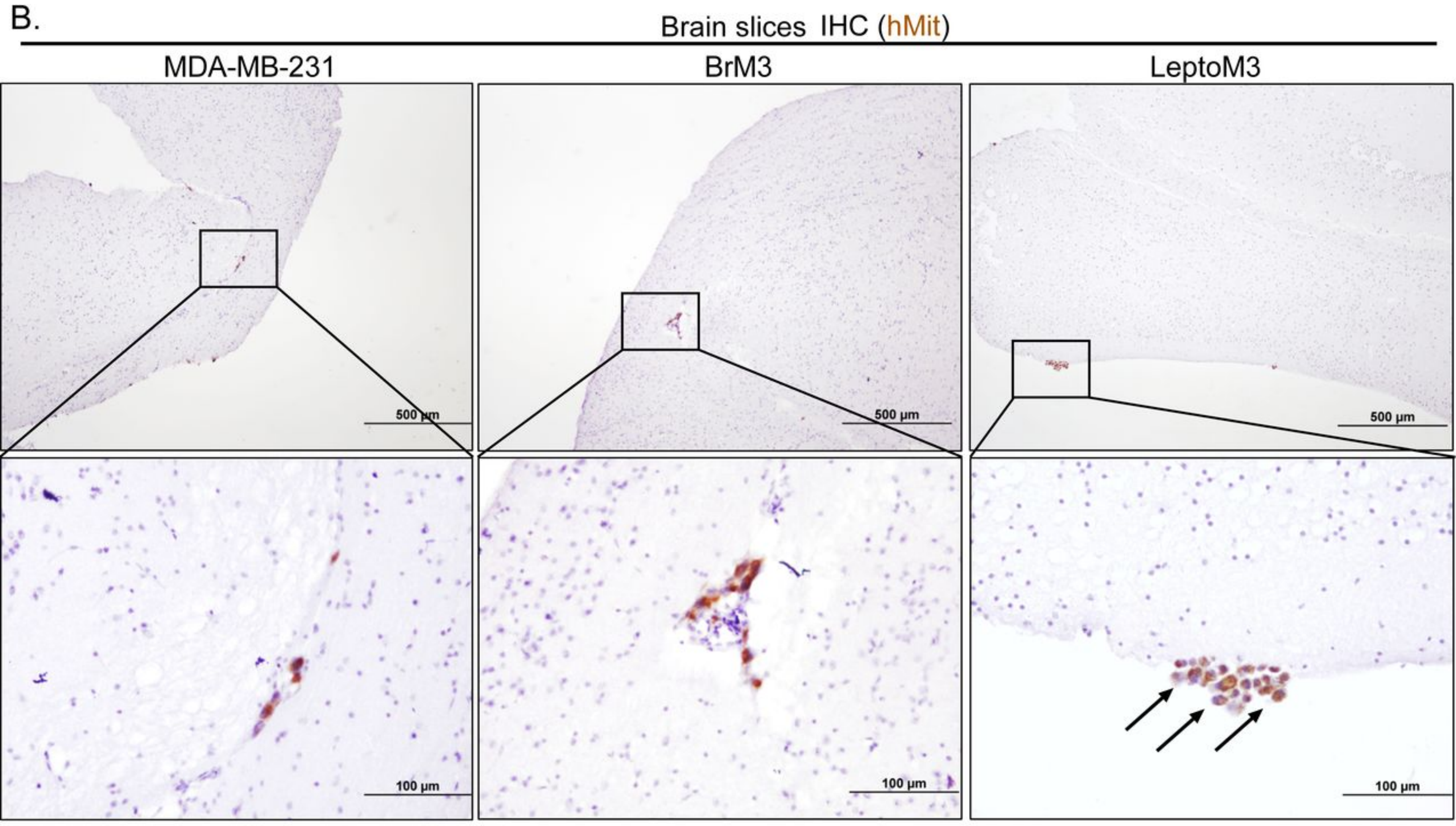

Figure S3 Pan et al.

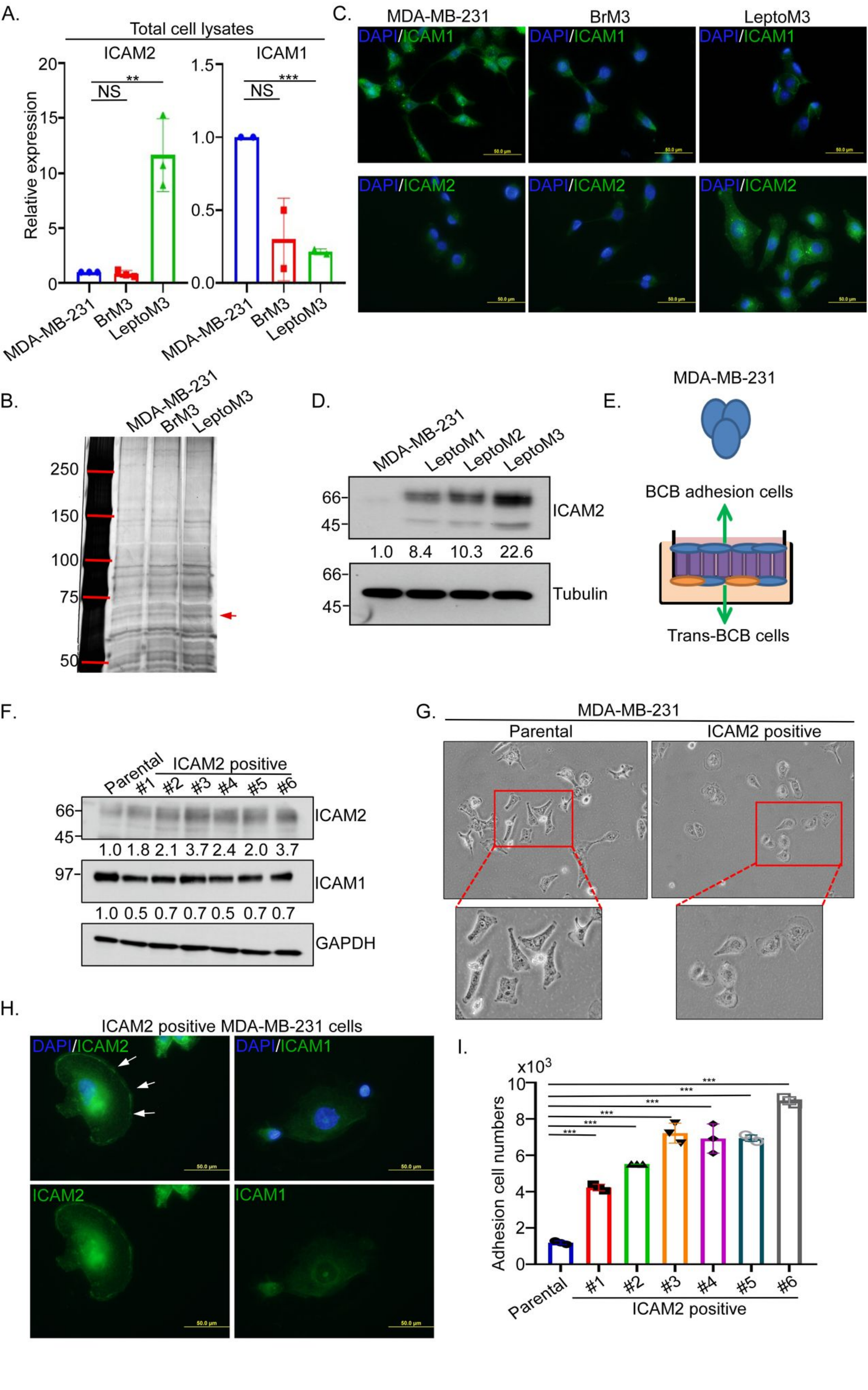

Figure S4 Pan et al.

A.

	LeptoM3 scramble (n=3)	LeptoM3 shICAM2 (n=3)
1 week	0%	0%
2 week	0%	0%
3 week	0%	0%
4 week	50%	0%
5 week	50%	0%
6 week	50%	0%
7 week	100 %	33.3%

B.

	MDA-MB-231 vector (n=3)	MDA-MB-231 ICAM2 (n=3)
1 week	0%	0%
2 week	0%	0%
3 week	0%	0%
4 week	0%	66.6%
5 week	0%	66.6%
6 week	0%	66.6%
7 week	0 %	66.6%

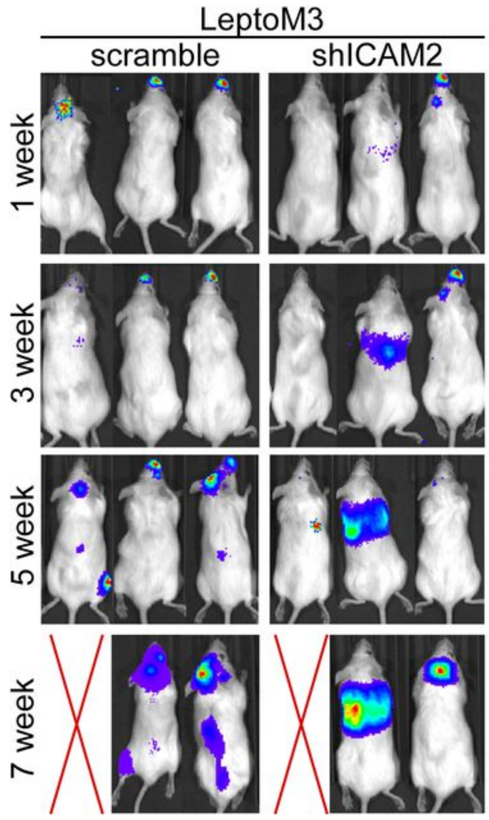

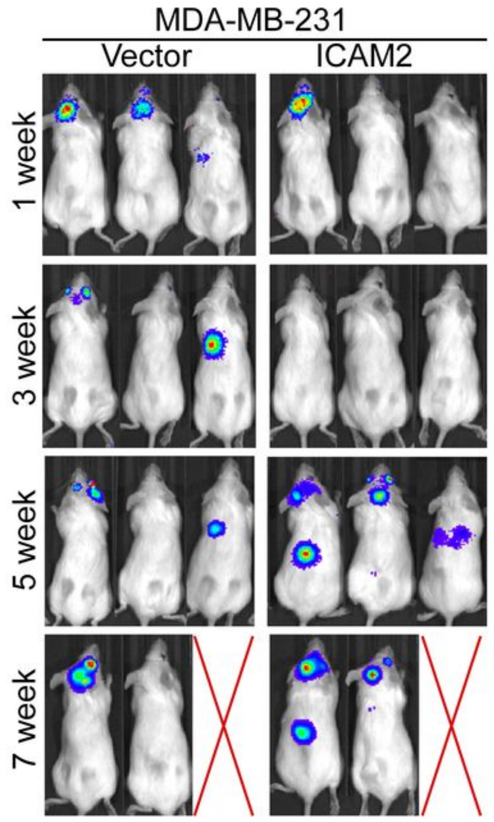

C.

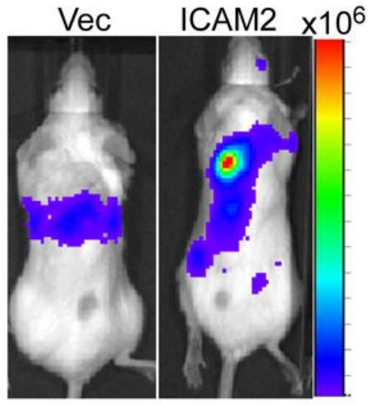

D.

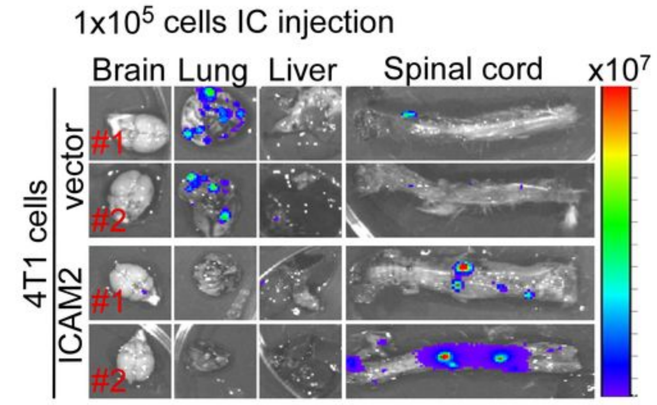

E.

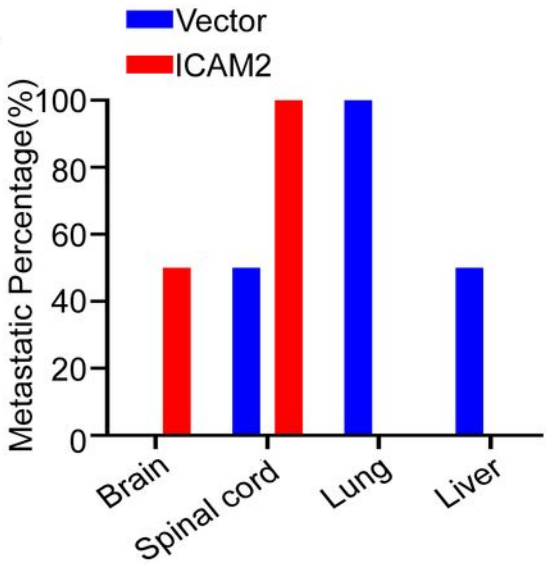

F.

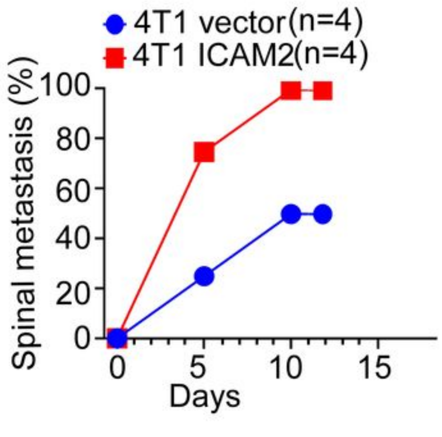

Figure S5 Pan et al.

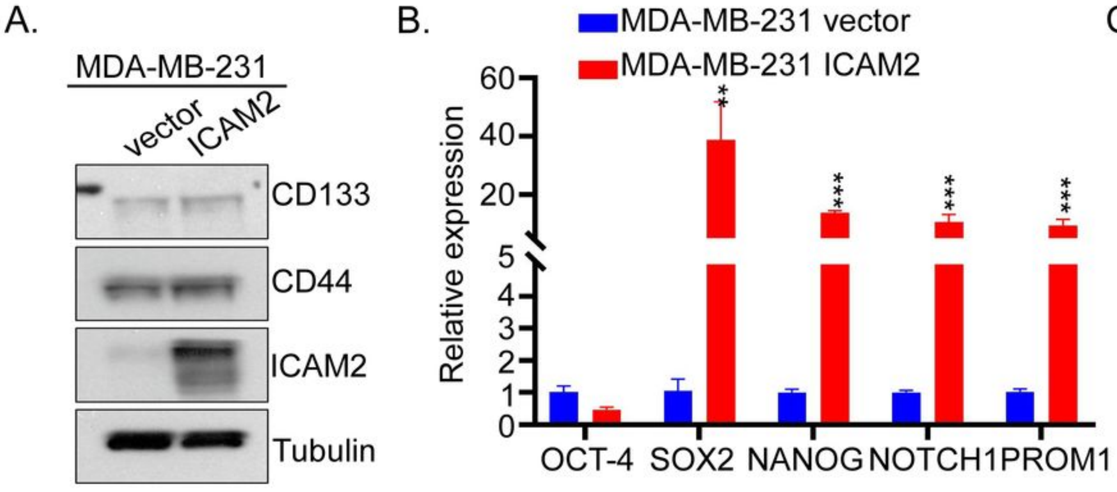

D.

MDA-MB-231

	1X10^5		1X10^3		1X10^2	
	**Vector**	**ICAM2**	**Vector**	**ICAM2**	**Vector**	**ICAM2**
1 week	4/4 100%	4/4 100%	0/6 0%	0/8 0%	0/4 0%	0/4 0%
2 week	4/4 100%	4/4 100%	0/6 0%	6/8 75%	0/4 0%	0/4 0%
3 week	4/4 100%	4/4 100%	2/6 33.3%	8/8 100%	0/4 0%	0/4 0%
4 week	4/4 100%	4/4 100%	2/6 33.3%	8/8 100%	0/4 0%	0/4 0%
5 week	4/4 100%	4/4 100%	3/6 50%	8/8 100%	0/4 0%	0/4 0%
6 week	4/4 100%	4/4 100%	3/6 50%	8/8 100%	0/4 0%	1/4 25%
7 week	4/4 100%	4/4 100%	3/6 50%	8/8 100%	0/4 0%	1/4 25%
8 week	4/4 100%	4/4 100%	3/6 50%	8/8 100%	0/4 0%	1/4 25%
9 week	4/4 100%	4/4 100%	3/6 50%	8/8 100%	0/4 0%	1/4 25%
10 week	4/4 100%	4/4 100%	3/6 50%	8/8 100%	0/4 0%	2/4 50%

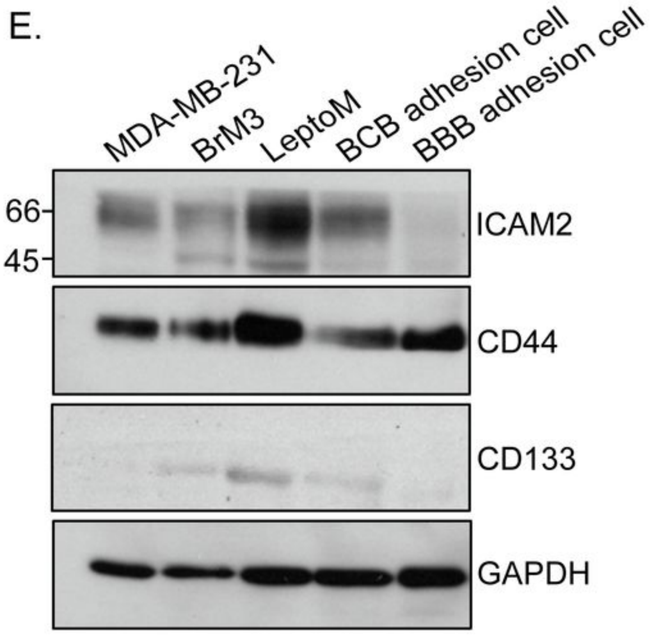

Figure S6 Pan et al.

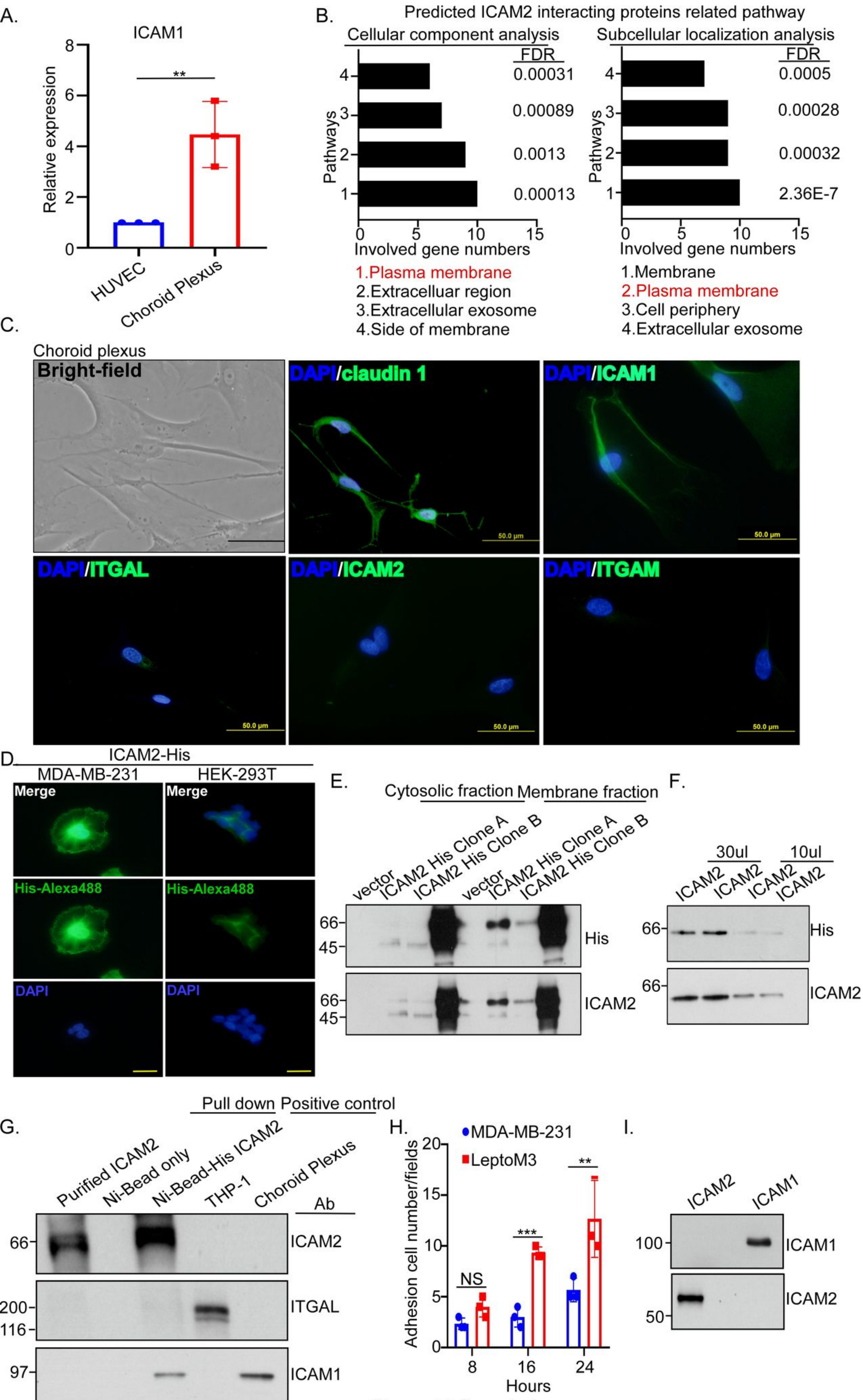

Figure S7 Pan et al.

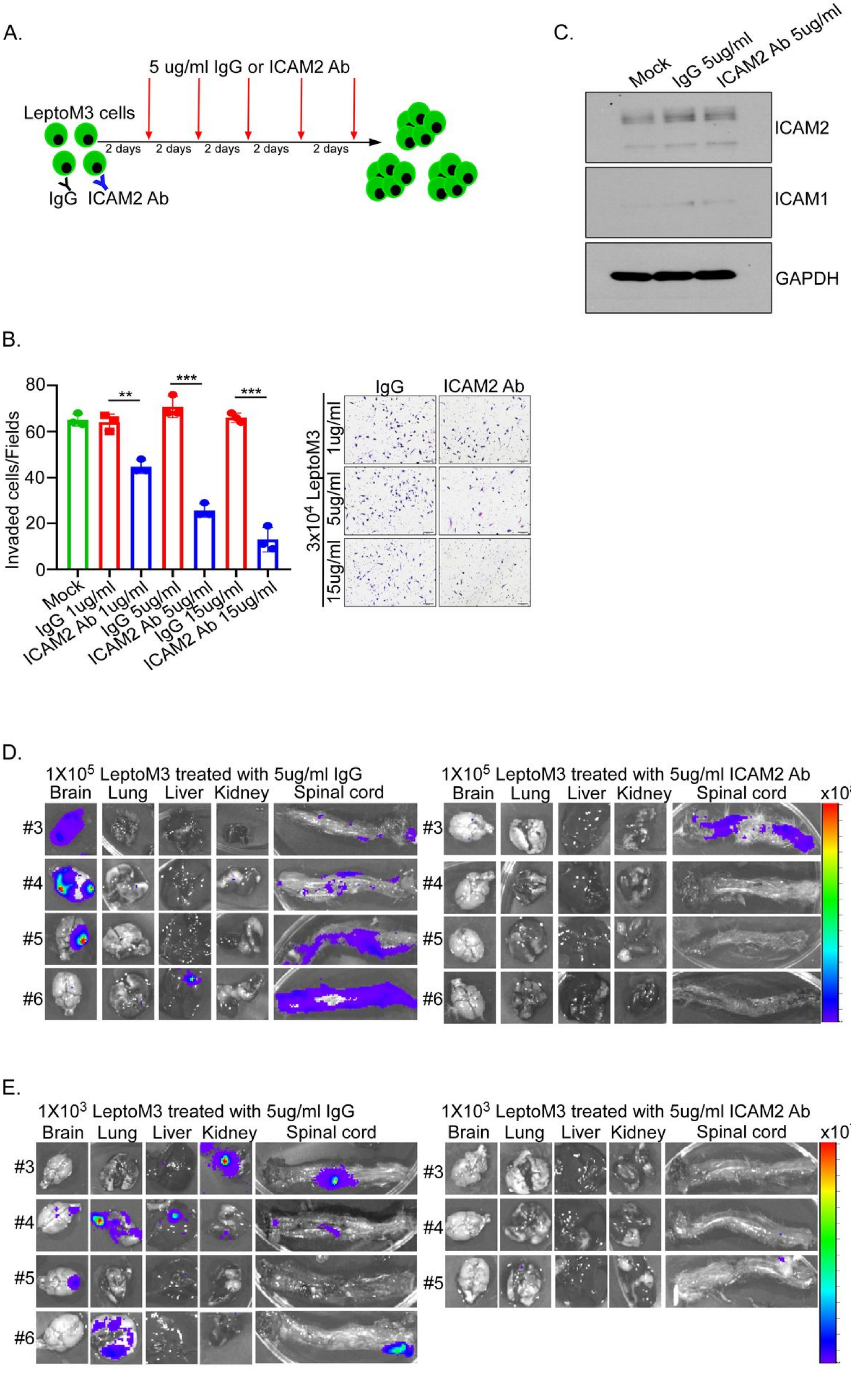

Figure S8 Pan et al.
